# Proteome specialization of anaerobic fungi during ruminal degradation of recalcitrant plant fiber

**DOI:** 10.1038/s41396-020-00769-x

**Published:** 2020-09-14

**Authors:** Live H. Hagen, Charles G. Brooke, Claire A. Shaw, Angela D. Norbeck, Hailan Piao, Magnus Ø. Arntzen, Heather M. Olson, Alex Copeland, Nancy Isern, Anil Shukla, Simon Roux, Vincent Lombard, Bernard Henrissat, Michelle A. O’Malley, Igor V. Grigoriev, Susannah G. Tringe, Roderick I. Mackie, Ljiljana Pasa-Tolic, Phillip B. Pope, Matthias Hess

**Affiliations:** 1 Faculty of Biotechnology, Chemistry and Food Science, Norwegian University of Life Sciences, Aas, Norway; 2 University of California, Davis, CA USA; 3 Pacific Northwest National Laboratory, Richland, WA USA; 4 Washington State University, Richland, WA USA; 5 Environmental Molecular Sciences Laboratory, Pacific Northwest National Laboratory, Richland, CA USA; 6 U.S. Department of Energy Joint Genome Institute, Lawrence Berkeley National Laboratory, Berkeley, CA USA; 7 CNRS, UMR 7257, Université Aix-Marseille, 13288 Marseille, France; 8 Institut National de la Recherche Agronomique, USC 1408 Architecture et Fonction des Macromolécules Biologiques, 13288 Marseille, France; 9 Department of Biological Sciences, King Abdulaziz University, Jeddah, 21589 Saudi Arabia; 10 Department of Chemical Engineering, University of California, Santa Barbara, CA USA; 11 Department of Plant and Microbial Biology, University of California, Berkeley, CA USA; 12 Department of Animal Science, University of Illinois, Urbana-Champaign, IL USA; 13 Faculty of Biosciences, Norwegian University of Life Sciences, Aas, Norway

**Keywords:** Microbiome, Fungi, Microbial ecology

## Abstract

The rumen harbors a complex microbial mixture of archaea, bacteria, protozoa, and fungi that efficiently breakdown plant biomass and its complex dietary carbohydrates into soluble sugars that can be fermented and subsequently converted into metabolites and nutrients utilized by the host animal. While rumen bacterial populations have been well documented, only a fraction of the rumen eukarya are taxonomically and functionally characterized, despite the recognition that they contribute to the cellulolytic phenotype of the rumen microbiota. To investigate how anaerobic fungi actively engage in digestion of recalcitrant fiber that is resistant to degradation, we resolved genome-centric metaproteome and metatranscriptome datasets generated from switchgrass samples incubated for 48 h in nylon bags within the rumen of cannulated dairy cows. Across a gene catalog covering anaerobic rumen bacteria, fungi and viruses, a significant portion of the detected proteins originated from fungal populations. Intriguingly, the carbohydrate-active enzyme (CAZyme) profile suggested a domain-specific functional specialization, with bacterial populations primarily engaged in the degradation of hemicelluloses, whereas fungi were inferred to target recalcitrant cellulose structures via the detection of a number of endo- and exo-acting enzymes belonging to the glycoside hydrolase (GH) family 5, 6, 8, and 48. Notably, members of the GH48 family were amongst the highest abundant CAZymes and detected representatives from this family also included dockerin domains that are associated with fungal cellulosomes. A eukaryote-selected metatranscriptome further reinforced the contribution of uncultured fungi in the ruminal degradation of recalcitrant fibers. These findings elucidate the intricate networks of in situ recalcitrant fiber deconstruction, and importantly, suggest that the anaerobic rumen fungi contribute a specific set of CAZymes that complement the enzyme repertoire provided by the specialized plant cell wall degrading rumen bacteria.

## Introduction

It has been estimated that there are ~1 billion domesticated ruminant animals [1] and numbers are predicted to increase further in order to provide food security for the growing human population [2]. The societal importance of ruminants has fueled global efforts to improve rumen function, which influences both animal health and nutrition. In particular, broadening the knowledge of the complex microbial interactions and the enzymatic machineries that are employed within the rumen microbiome is thought to provide means to efficiently optimize feed conversion, and ultimately the productivity and well-being of the host animal.

One of the major functions mediated by the rumen microbiome is to catalyze the breakdown of plant carbon into short-chain fatty acids that can be metabolized by the host animal. To facilitate the degradation of complex plant carbohydrates, the rumen microbiome encodes a rich repertoire of carbohydrate-active enzymes (CAZymes). This group of CAZymes is categorized further into different classes and families, which include carbohydrate-binding modules (CBMs), carbohydrate esterases (CEs), glycoside hydrolases (GHs), glycosyltransferases (GTs), and polysaccharide lyases (PLs) [3]. Previous studies have mostly been dedicated to CAZymes from rumen bacteria, although it is becoming increasingly clear that fungi and viruses also possess key roles in the carbon turnover within the rumen [4, 5]. Over the last decade, targeted efforts to isolate and cultivate novel rumen microorganisms have resulted in a more detailed understanding of the physiology of anaerobic rumen archaea and bacteria and their contribution to the overall function of the rumen ecosystem [6]. An enhanced focus on isolation, cultivation and characterization of novel anaerobic rumen fungi and protozoa has also provided important insight into their lifestyle and enzymatic capacity [4, 7, 8], although their quantitative metabolic contributions to the greater rumen ecosystem are still not fully understood.

Enumerating anaerobic rumen fungi is challenging, mainly due to their different life stages and their growth within plant fragments as well as sub-optimal DNA extraction and molecular methods to recover their genomic information [9–11]. Reported counts of fungal cells vary greatly between studies, with numbers ranging between 10^3^ and 10^6^ cells/ml of rumen fluid [12–14]. To date, only a total of 18 genera, all belonging to the early-branching phylum Neocallimastigomycota, have been described [4, 15–17], although culture independent studies have suggested that this only represents half of the anaerobic fungal population that exist in the rumen ecosystem [15, 18]. Genomes obtained from representatives of this phylum have been recognized to encode a large number of biomass-degrading enzymes and it is becoming evident that these currently still understudied organisms play a key role in the anaerobic digestion of complex plant carbohydrates [4, 7, 17]. The impact of fungi in the rumen ecosystem was already demonstrated in the early 1990s by Gordon and Phillips who reported a significant decrease in fiber digestion within the rumen after anaerobic fungi had been removed by the administration of fungicides [19]. The importance of rumen fungi for biomass degradation has since then been supported by in vivo studies [20–22], and recently reinforced in transcriptome studies revealing that the fungi express a range of CAZymes when grown on different carbon sources [23, 24]. Although enzymes of fungal origin have been regularly explored for their remarkable capacity to degrade lignocellulosic fiber [10, 25, 26], their functional role in native anaerobic habitats and within the biomass-degrading enzyme repertoire of the rumen microbiome remains unclear. Thus, we lack a complete understanding of their biology and their contribution to the function and health of the rumen ecosystems.

To fill this knowledge gap, we utilized a genome-centric metaproteome approach to investigate the distinct role of the fungal population during the biomass-degradation process in the rumen. Our experiments were designed to target populations actively degrading recalcitrant fibers that resisted initial stages of microbial colonization and digestion. Specifically, metaproteomic data were interrogated using a database constructed from five available rumen fungal isolates [4] in addition to genomes and metagenome-assembled genomes (MAGs) of cultured and uncultured rumen bacteria, respectively. To further explore the activity of uncultured fungi, we performed a second metaproteomic search against a database generated from polyadenylated mRNA extracted from rumen-incubated switchgrass. Combining data from these various layers of the rumen microbiome enabled us to generate new insights into the functional role of anaerobic rumen fungi, expanding our holistic understanding of plant-fiber decomposition in the rumen ecosystem.

## Material and methods

### Rumen incubation and sample collection

Air-dried switchgrass was milled to pass through a 2 mm sieve and weighed into individual in situ nylon bags (50 μm pores; Ankom Technology, Macedon, NY, USA). To enrich for lignocellulolytic microorganisms, the Nylon bags, each containing 5 g of air-dried switchgrass, were placed in the rumen of two cannulated Holstein-Friesians cows (adults) as described previously [27]. To capture microbes involved the second phase of switchgrass degradation, which last from ~6 h until ~72 h after the recalcitrant plant material has been introduced into the rumen system [28], switchgrass-filled nylon bags were retrieved from the cow’s rumen after 48 h, washed immediately with PBS buffer (pH7) to remove loosely adherent microbes, frozen immediately in liquid nitrogen and transported to the laboratory. Samples were stored at −80 °C until protein and RNA extraction was performed. All animal procedures were performed in accordance with the Institution of Animal Care and Use Committee (IACUC) at the University of Illinois, under protocol number #06081.

### Construction of a rumen-specific reference database (RUS-refDB)

To resolve the roles of the fungal, bacterial and viral populations, we designed a customized RUmen-Specific reference DataBase (hereby referred to as ‘RUS-refDB’). During initial analysis, preliminary databases were constructed from a comprehensive collection of available genomes from the Hungate1000 collection and rumen-derived metagenomes. However, as many search engines, including Andromeda [29] utilized by MaxQuant [30], rely on a target-decoy approach to control the false discovery rate (FDR), large and unspecific databases results in a vastly increased search space and sequence similarity that makes the FDR-control challenging [31]. To overcome this, the final RUS-refDB was constructed to be as dedicated to the metaproteome as possible, i.e., consisting of MAGs and a virome recovered from an equivalent experimental design [27, 32]. The protein sequence database was further augmented with genomes of isolated microorganisms known to be prevalent in the rumen, yet lacking from the high-quality MAG collection.

In summary, the collection of protein sequences from rumen associated microorganisms was generated from a total of 122 microbial genomes (bacterial MAGs and isolates of fungi, bacteria, and archaea) and 931 metagenome-assembled viral scaffolds (MAVs). The bacterial MAGs originated from a metagenome recovered from rumen-incubated switchgrass enrichments from Hess et al. (2011) as well as from a recent re-assembly of the Hess et al. metagenome published by Parks et al. (2017). Genome redundancy was reduced by removing genomes with an amino-acid identity (AAI) > 99% (CompareM v.0.0.13; https://github.com/dparks1134/CompareM), of which the MAGs with the highest quality (CheckM [33] v.1.0.18) were kept for downstream analysis. This resulted in a non-redundant catalog of high-quality switchgrass-enriched rumen MAGs, composed of 7 and 96 MAGs from Hess et al. (2011) and Donovan et al. (2017), respectively. Predicted genes (GeneMark [34]) in MAVs, previously recovered from the same rumen metagenome [27, 32], were also included in the database. While not elaborated in the main results, key findings related to the viral population can be found in Supplementary Material (Figs. S1, S2 and Text S1). To account for additional prokaryotic rumen populations that are well-known to be metabolically active and engage in major metabolic function [22, 35, 36], we further selected 11 genomes from GeneBank and the Hungate1000 project [6]: *Bacillus licheniformis* strain VTM3R78, *Butyrivibrio fibrisolvens* strain AR40, *Prevotella ruminicola* strain BPI-162 and strain KHT3, *P. brevis* strain P6B11, *P. bryantii* strain KHPX14, *Ruminococcus albus* strain KH2T6, *R. flavefaciens* strain Y1, strain MA2007, strain ND2009 and strain SAb67. We supplemented these core genomes with the genome of *Fibrobacter succinogenes* S85 [37] and *Methanobrevibacter ruminantium* M1 [38], both known to play a significant role in proper rumen function. To specifically determine the functional roles of anaerobic fungi in the rumen, we also included protein sequences from the genomes of the five cultivated anaerobic fungi available at the time; *Anaeromyces robustus* [4, 39], *Neocallimastix californiae* G1 [4], *Orpinomyces* sp. [17] (proposed to be re-classified as *Pecoramyces ruminantium* C1A [16]), *Piromyces* sp. E2 [4] and *Piromyces finnis* [4, 39], downloaded from MycoCosm [40] (https://mycocosm.jgi.doe.gov). A summary of the MAGs, MAVSs, and genomes from isolates that made up RUS-refDB is provided in Supplementary Table S1. This sequence collection was further used as a comprehensive reference database for mapping of the metaproteome data, as described below.

### Phylogenetic tree

For the phylogenetic tree, we searched each genome and MAG included in the RUS-refDB for 21 ribosomal proteins (i.e., L1, L3, L4, L5, L6, L11, L13, L18, L22, L24, S2, S5, S8, S9, S10, S11, S12, S13, S15, S17, and S19). The resulting ribosomal protein sequences were aligned separately using MUSCLE [41] v3.8.31 and manually checked for duplication and misaligned sequences. For further alignment clean-up, GBlocks [42] v.0.91b with a relaxed selection of blocks (settings: -b2 = 50 -b3 = 20 -b4 = 2 -b5 = a) was employed. The alignments were then concatenated using catfasta2phyml.pl (https://github.com/nylander/catfasta2phyml) with the parameter ‘-c’ to replace missing ribosomal proteins with gaps (-). The concatenated alignment was initially tested using MEGA-X [43] to select the best-fit model for protein substitution, while the maximum likelihood phylogenetic tree was constructed using RAxML [44] v.8.2.12 (raxmlHPC-SSE3 under PROTGAMMA with WAG substitution matrix and 100 rapid bootstrap inferences). One MAG (UBA1267) was not included in the ribosomal protein tree due to undetermined values. This tree was then re-built from a separate alignment including two ribosomal proteins (L3 and S9) from the five rumen fungi included in RUS-refDB, and finally visualized using iTol [45]. A complete version of the tree is available in Newick format as Supplementary Data S1. To obtain an overview of the microbial taxa associated with our detected proteins, we decorated the generated phylogenetic tree with the numerical detection of proteins for each taxon in both the switchgrass fiber fraction and rumen fluid.

### Metaproteomics—protein extraction and mass spectrometry

Protein extraction and mass spectrometry were performed on rumen-incubated switchgrass and bulk rumen fluid as described previously in Naas et al. (2018). The obtained MS/MS scans were subsequently analyzed using MaxQuant [30] v.1.6.0.13. Proteins quantified using the MaxLFQ [46] algorithm implemented in MaxQuant, and the peptides were identified by searching the MS/MS datasets against the reference databases as described in Supplementary Text S2. All identifications were filtered in order to achieve a protein FDR of 1% using the target-decoy strategy. The software Perseus version 1.6.0.7 [47] was used for downstream interpretation and quality filtering, including removal of decoy database hits, hits only identified by site and contaminants (see Text S2 for details). Finally, at least one unique peptide per protein was required for a protein to be considered as valid. Perseus was also used to perform correlation analysis of Log_2_(LFQ) values between the two biological replicates.

### Metatranscriptomics—total RNA extraction and Poly(A) mRNA purification

Poly(A) mRNA, representing transcripts of eukaryotic origin was isolated from total RNA as described previously [48]. Cleaned reads were combined and the metatransciptome was assembled using MEGAHIT [49] v.0.2.0. After sample preparation, sequencing, filtering, and assembly as described in Supplementary Text S2, the obtained transcripts were mapped against the assembled genome of each of the five fungal species represented in RUS-refDB (i.e., *Anaeromyces robustus*, *Neocallimastix californiae* G1, *Orpinomyces* sp., *Piromyces* sp. E2 and *Piromyces finnis)* using BWA-MEM [50], and those aligned to genomes were excluded from downstream analysis. In addition, contigs shorter than 1 kb were removed from the dataset. TransDecoder v.2.0.1 (http://transdecoder.sf.net) with default settings was used to identify open reading frames (ORFs) within the transcripts and the resulting sequences (256 232 ORFs) was used as a eukaryote-associated database (hereby referred to as ‘MT-eukDB’). The MS scans retrieved from the extracted metaproteome was then searched against MT-eukDB in the same manner as described previously for RUS-refDB.

### Functional annotation and metabolic reconstruction

All protein sequences included in the RUS-refDB and MT-eukDB were functionally annotated using InterProScan5 [51] v.5.25–64, comprising a search against the Pfam and CDD databases, Gene Ontology (GO) annotation and mapping to KEGG pathway information. CAZymes in both databases were additionally annotated using the CAZy annotation pipeline [52]. This functional annotation information was added to the detected protein groups in Perseus, and manually searched for specific metabolism. To ensure high confidence results in the reported CAZyme, the protein had to be detected in both biological replicates (i.e., in both cows) in at least one of the two microhabitats (i.e., switchgrass fiber and rumen fluid). Protein groups not fulfilling these criteria were omitted from the main results. For the reconstruction of active pathways involving monosaccharide degradation and fermentation, we scanned the detected protein in each of the annotated genomes and MAGs for signature pfam IDs, and further validated its function using its Interpro, CDD, and GO annotation. A complete or nearly complete set of pathway genes was needed to be turned on for a genome to be considered as actively involved in a respective metabolism. The protein detection levels of each protein group are reported as the average Log_2_(LFQ) for each biological replicate, which enables the quantification of the active metabolic function of the keystone rumen populations. Heat maps were generated with the ggplots package heatmap.2 in RStudio [53] v.3.6.1. While only the protein profile for switchgrass fiber is displayed in the CAZyme and metabolic-specific heat maps in order to reduce complexity, corresponding heat maps including rumen fluid are available in the Supplementary Material (Figs. S3 and S4). Furthermore, as there might be ambiguity in the protein-to-organism inference (i.e., the protein might origin from a closely related strain not present in our database) the term “well aligned proteins” was introduced to denote protein-affiliation to specific species in the database.

## Results and discussion

### Taxonomic origin of proteins involved in rumen biomass-degradation

**Fig. 1:**
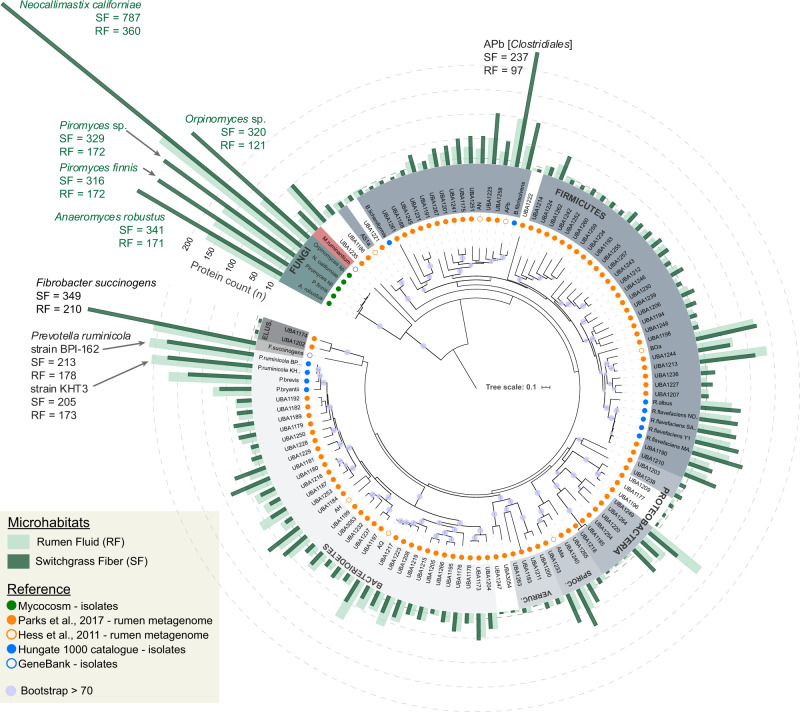
Concatenated ribosomal protein tree of the genomes and metagenome-assembled genomes (MAGs) included in RUS-refDB. Phylum-level groups are colored in shades of gray (bacteria), red (archaea) and green (fungi), and labeled inside the circle (Spiroc. Spirochaetes, Verruc. Verrucomicrobia, Elus. Elusimicrobia). MAGs/clades with uncertain taxa have white background. Circles centered on the nodes indicates a bootstrap support >70, out of 100 bootstraps. Circles at the end of each node are color coded by the metagenome dataset or genome collection each MAG/genome in RUS-refDB originated from, as indicated in the top left legend. The number of different proteins detected from the samples in the switchgrass fiber fraction (dark green) and rumen fluid (light green) are specified by bars surrounding the tree. In cases where a protein group consisted of two or more homologues protein identifications, each protein match is considered. The viral scaffolds, not included in the tree, had 56 and 62 proteins detected in switchgrass fiber and rumen fluid respectively (Fig. S1). Numerical protein detection can be found in Table S1. A complete version of this tree is available in Newick format as Supplementary Data S1.

Mapping the protein scans from switchgrass fiber and rumen fluid against the RUS-refDB resulted in the identification of a total of 4673 protein groups, and a strong positive correlation (Pearson correlation *r* > 0.8) of the two biological replicates (cow 1 and cow 2) was obtained (Supplementary Fig. S5). This level of identification was similar to previous metaproteomic studies of anaerobic digestion consortia that used a sample-specific database [54], validating our database design. Interestingly, the (meta)genome-resolved metaproteome revealed that a high fraction of detected proteins within our metaproteome were of fungal origin (Fig. 1, numerical detection of proteins can be found in Supplementary Table S2). Within the five anaerobic rumen fungi, we observed between 316 and 787 proteins that aligned well to proteins predicted from the genomes of *Piromyces finnis* and *Neocallimastix californiae*, respectively. This exceeds the number of proteins detected from any of the investigated prokaryotes included in this study, and likely reflects the fundamental functional role that fungi hold in ruminants during degradation of recalcitrant cellulosic material. Moreover, the metaproteomics data also revealed a higher level of protein grouping across the fungal genomes due to homologous proteins, suggesting that there are conserved features of the fungal genomes that have been sequenced to date. Many of the corresponding protein-coding genes were also replicated within each fungal genome, demonstrating that individual rumen fungi hold several sets of functionally important genes. Despite a reportedly high degree of horizontal gene transfer (HGT) in the rumen microbiome [4, 55, 56], only a few detected proteins mapped to both fungi and prokaryotes (Supplementary Data S2; ‘Major protein IDs’), suggesting that the overall sequences of these particular enzymes are evolutionary divergent across these two kingdoms.

The bacterial portion of the RUS-refDB was mostly comprised of genomes belonging to the Firmicutes and Bacteroidetes phyla, of which species belonging to the *Ruminococcus* and *Prevotella* accounted for a large fraction of the detectable proteins (Fig. 1). A high number of detected proteins also aligned well to the genome of *Butyrivibrio*, emphasizing the significance of this group in biomass-degradation and conversion within the rumen. Within this clade, ‘APb’, a MAG closely related to *Butyrivibrio fibrisolvens*, showed the highest number of detected proteins from an as-yet uncultured prokaryote (switchgrass: 237; rumen fluid: 97). The well-studied fibrolytic bacteria *Fibrobacter succinogenes* represented the bacterial species with the highest number of detected proteins (switchgrass: 349; rumen fluid: 210), followed by two strains of *Prevotella ruminicola* (ranging from 173 to 213 proteins, of which the majority of the detection proteins were homologues of the two strains) and *P. brevis* (switchgrass: 129; rumen fluid: 168), highlighting their overall importance in the carbohydrate metabolisms in the rumen. This is consistent with previous studies involving functional analyses of the rumen microbiome, demonstrating that a majority of the plant cell wall polysaccharide degradation is carried out by species related to *Fibrobacter*, *Ruminococcus,* and *Prevotella* [21, 22, 57]. Although our metaproteome data suggested that these aforementioned characterized prokaryotes were amongst the most active (i.e., highest numbers of detected proteins), a significant fraction of the protein groups mapped to MAGs representing uncultured and uncharacterized taxa. This included MAGs classified within the *Bacteroidetes* phyla, such as UBA1181 previously described by Naas et al. [58], a clade consisting of the *Spirochaetes*-assigned MAG ‘AMa’, UBA1233 and UBA1240, in additional to a *Proteobacteria*-clade (UBA1249, UBA1220 and UBA1264). This reiterates that a considerable fraction of the bacterial rumen microbiome remains to be explored and characterized before a holistic and truly advanced understanding of the role of rumen bacteria is achieved.

### Metaproteome-generated CAZyme profile indicates compartmentalized niches amongst fungal and bacterial populations

**Fig. 2:**
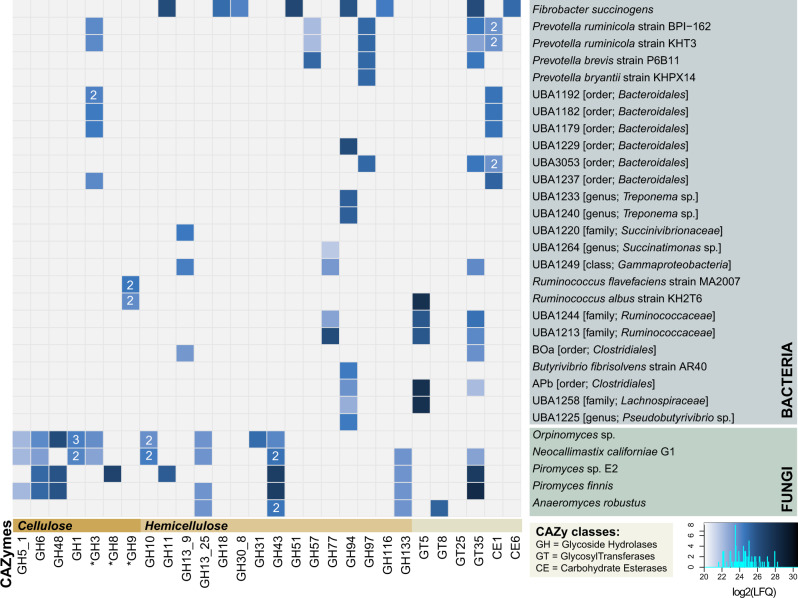
CAZyme profile from each predicted source organism in RUS-refDB, displaying the detected proteins associated with the milled switchgrass. Here, we focused only on CAZymes detected in both animals to achieve high confidence detection of the active key populations. CAZy families that might possess activity against cellulose and hemicellulose are indicated with an asterisk. The colors in the heat map indicates the protein detection levels of each protein group reported as the average Log_2_(LFQ)-scores for the biological replicates, where a light blue color is low detection while darker is high protein detection. Some of the source organisms encompassed more than one detected variant of GH1, GH3, GH43, and CE1. For these cases, the protein detection is reported as the average Log_2_(LFQ)-score and number of variants are indicated within the heat map. A corresponding figure showing the detection level for all variants separately, as well as the detection in the rumen fluid microhabitat is provided in Supplementary Fig. S3.

The efficiency of the rumen microbiome in breaking down the complex cell wall of plants is due to the orchestrated synthesis, degradation, and modification of glycosidic bonds by an intricate mixture of microorganisms and their CAZymes. Crystalline cellulose is often degraded through a synergistic mechanism between endo- and exo-acting CAZymes targeting the glycosidic bonds within or at the ends of the polysaccharide, respectively. Notably, the exo-acting cellulases with highest protein abundance (measured by Label-Free Quantification (LFQ)) in our dataset, such as GH6 and GH48, appeared to come nearly exclusively from the rumen fungi (Fig. 2). Moreover, GH48, aligning well to predicted protein sequences from both *Orpinomyces* sp. and the two *Piromyces* species, had the highest LFQ-level of all cellulases detected in the fiber adherent microhabitat. In contrast, GH48 was only detected at lower levels in the rumen fluid (Supplementary Fig. S3). Family GH48 consists of endo-processive cellulases with favored activity on amorphous or crystalline cellulose and play a key role in the decomposition of recalcitrant plant fiber [59]. Although members of this family also exist as free enzymes, they are often amongst the most abundant CAZymes in cellulosome complexes [60, 61]. Accordingly, nearly all GH48 enzymes detected in our metaproteome contained cellulosome signature Type-II dockerin domains in tandem repeats (Supplementary Table S2 and Text S1), strongly suggesting that anaerobic fungi employ GH48 in multi-modular enzymatic complexes to efficiently degrade crystalline cellulose. This observation is consistent with the powerful degradation activity of fungal multi-modular complexes previously demonstrated by Haitjema et al. (2014).

While GH48 have been absent or only detected at very low levels in previous rumen metagenomes [27, 62], members of this GH family have been observed in rumen metatranscriptomes from mixed rumen populations, reportingly expressed by *Ruminococcus* and rumen fungi [21, 22, 63]. It is also worth noting that while members of the GH48 family were the most abundant CAZymes affiliated to protein sequences of *Orpinomyces* sp. origin, other detected proteins belonging to the GH families GH1, GH3, GH5_1, and GH6 encompassing cellulases, also aligned to the proteome of this fungus. In contrast to the elevated number of mapped proteins (Fig. 1), CAZymes predicted from the genome of *N. californiae* contributed at lower detection levels than its fungal companions, albeit its CAZyme profile covered proteins with a wide range of substrate specificity including both cellulose (i.e., β-glucosidases, GH1 and GH3; endoglucanases, GH5_1 and GH6), starch (amylase and amyloglucosidases, GH13_25-GH133) and hemicellulose (xylanases, GH10 and GH43). CAZymes inferred in the conversion of starch and hemicellulose also aligned well to the four other fungal reference genomes, with elevated level of xylanases belonging to the GH43 family (Fig. 2, Supplementary Data S2). Members of GH43 may also be active on arabinan components in pectin [64, 65], another major component of the plant cell wall. Yet, although RUS-refDB contained a relatively large portion (*n* = 314) of PLs, including PL1 (pectate lyase/exo-pectate lyase/pectin lyase), PL3 (pectate lyase), PL4 (rhamnogalacturonan lyase), PL9 (pectate lyase/exopolygalacturonate lyase) and PL11 (rhamnogalacturonan lyase), none of these were detected in the metaproteome. Likewise, no key enzymes associated with lignin degradation could be detected, possibly reflecting that enzymes involved in degradation of pectin and lignin primarily occur at different time points of rumen-incubation.

Despite the cellulose-degrading reputation of *F. succinogenes*, the detected CAZymes were predominately involved in soluble glucans and/or hemicellulose degradation (Fig. 2), with representatives belonging to the family of GH11, GH51, and GH94 amongst the most abundant GHs. In addition to *F. succinogenes*, *R. albus* and *R. flavefaciens* have also been repeatedly shown to contribute many of the required CAZymes for biomass-degradation in the rumen [57, 66–68]. Indeed, GH9 endoglucanases, representing a CAZyme family capable of hydrolyzing β 1 → 4 glyosidic bonds in cellulose, were detected in the proteome of both *R. albus* and *R. flavefaciens*. Members of the previously mentioned GH48-family, that suggested *Ruminococcus* sp. as key to cellulose degradation [69, 70], however, were not detected.

Overall, it appears that within our experimental constraints (switchgrass incubated for 48 h), bacterial populations contributed CAZymes that primarily modified non-cellulosic plant carbohydrates. Moreover, the number of detected CAZymes, as well as the protein detection level for specific GHs, was generally higher in the switchgrass-associated microhabitat than in the rumen fluid (Supplementary Figs. S1 and S3). This was particularly true for the fungal population, whereas the saccharolytic bacterial counterparts seemed less designated to any particular microhabitat. Importantly, these observations support the assumptions that rumen fungal populations are specialized toward the recalcitrant share of the fiber deconstruction. It should nevertheless be emphasized that the metaproteome data analyzed here represents only a snapshot of the community metabolism, and that the protein profiles of different rumen populations (e.g., *Ruminococcus*-affiliated cellulases) most likely have undergone temporal transformations in the time period between the plant material being introduced into the rumen environment and when samples were collected for our omics analyses  [20, 71].

### Metatranscriptomics of as-yet uncultured populations support predictions that fungi are active lignocellulose-degraders within the rumen ecosystem

**Fig. 3:**
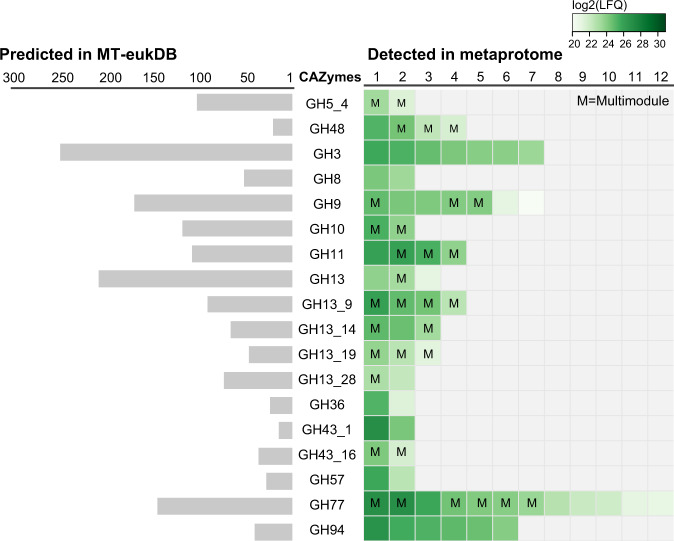
Visualization of the number of predicted genes annotated to specific GH families in MT-eukDB (left) and those detected when searching MT-eukDB against the metaproteome (right). Only CAZymes detected in both animals in at least one of the microhabitats are included to achieve high confidence detection. The colors of the squares in the right panel indicates the protein detection level for each individual protein, reported as the average log_2_(LFQ) of the biological replicates, where light green represents low detection level while darker green is high protein detection level. While this figure only shows those detected in the milled switchgrass, a comprehensive table of all CAZymes detected in both switchgrass and rumen fluid can be found in Supplementary Data S3. This also includes details regarding proteins with multiple CAZyme modules (indicated with an ‘M’).

As a limited number of genomes from anaerobic fungi are currently publicly available, we expected that our RUS-refDB would only represent a fraction of the anaerobic fungal population in the rumen. In an attempt to overcome this constraint, we constructed a complementary database based on a eukaryote-derived metatranscriptome (‘MT-eukDB’), originating from 423,409,432 raw Illumina reads (~63.5 Gbps) recovered from the community that colonized milled switchgrass during rumen-incubation (Supplementary Table S3). After quality filtering and assembly of the raw reads, we identified a total of 4,581,844 expressed genes for which 4,550,231 (99.31%) were predicted to encode proteins. The assembled metatranscriptome was filtered against the genome of the cultured fungi before the MT-eukDB was used as a database to identify peptides derived from uncultured rumen fungi within the generated metaproteome data. As with the proteomes of the five fungal isolates, this mapping effort revealed a repertoire consisting of CAZymes belonging to the families of GH3, GH8, GH9, GH10, GH11, GH13 (subfamilies), GH36, and GH48 (Fig. 3, Supplementary Data S3). While only detected amongst the bacterial population in RUS_refDB, CAZymes were additionally assigned to the families of GH77 and GH94. Similar to RUS-refDB, a higher detection level was observed for several of the CAZymes affiliated to the switchgrass fiber compared to rumen fluid, including GH3, GH94 and two of the detected GH48s (Supplementary Data S3). As fragments believed to be of bacterial origin were observed in MT-eukDB, we searched the detected gene sequences of the GH48 representatives against the non-redundant (nr) protein database from NCBI (http://www.ncbi.nlm.nih.gov/) using BLASTP [72] (May 23rd, 2020), which confirmed that these protein sequences best resembled GH48 of *Ruminococcus* sp. (sequence similarity ranging from 64 to 97% identity. BLAST results can be found in Supplementary Data S3, and GH48 protein sequences are available in Text S3). Despite this seemingly conflicting result, this was not unexpected, given the scarcity of characterized fungal GH48s and the documented frequency of inter-kingdom HGT of catalytic domains in gut ecosystems [4, 73], especially for GH48s [74]. Nevertheless, while we postulate that these active GH48s originate from anaerobic fungi, likely achieved through HGT events, we cannot exclude that bacterial transcripts are present in the metatranscriptome.

### Toward a holistic understanding of the functional roles of rumen populations

**Fig. 4:**
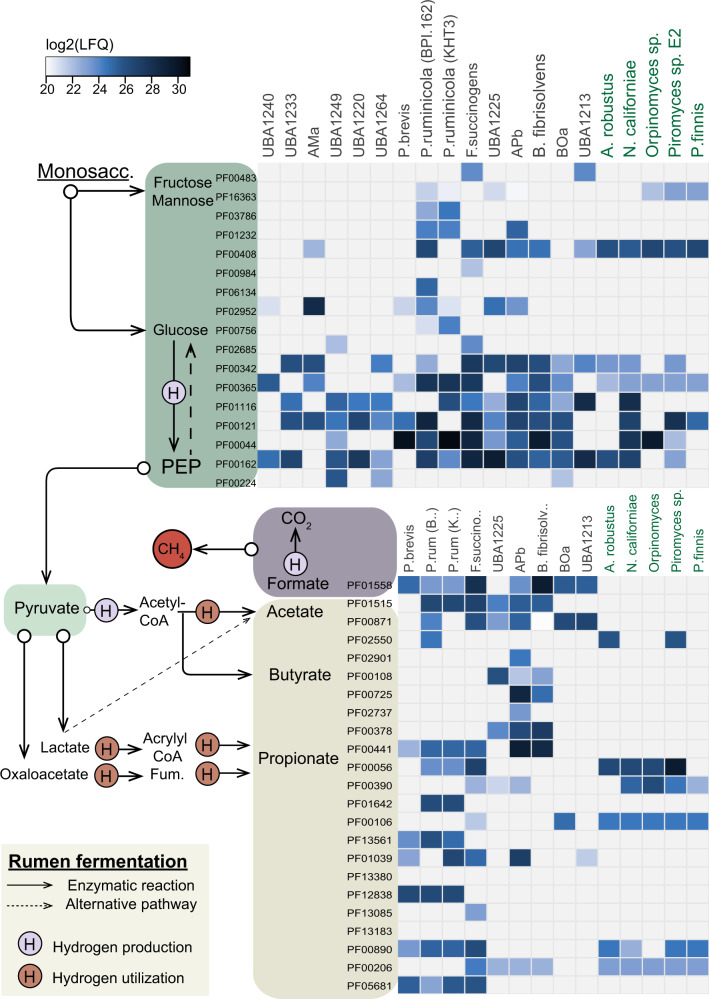
Metabolic reconstruction of key players intermediate rumen fermentation as determined in this study. The heat map shows the detection of proteins associated to main metabolic pathways (listed as pfam IDs) found in the most active genomes/MAGs (indicated on the top: Anasp *Anaeromyces robustus,* Pirfi *Piromyces finnis, PirE2 Piromyces* sp. E2, Neosp *Neocallimastix californiae*, Orpsp *Orpinomyces* sp.) in RUS-refDB. The colors in the heat map indicates the protein detection levels reported as the average log_2_(LFQ)-scores for each biological replicate, where light blue represent lower detection levels while darker blue is high protein detection. Only the proteins from the switchgrass are included in the current figure, while a corresponding figure including protein detection from both switchgrass and rumen fluid is available in Supplementary Fig. S4. A comprehensive table including proteins detected in all MAGs/genomes included in RUS-refDB, proteins associated to the rumen fluid and the functional categorization of the pfam IDs can be found in Supplementary Data S2.

The initial degradation of complex plant fiber makes the carbon pool available for downstream metabolism that encompasses the intricate microbial food web within the rumen, ultimately providing access to otherwise inaccessible nutrients to the host. Concurrent with previous rumen metaproteome and metatranscriptome studies [20, 75], our analysis revealed that the prokaryotic population in the rumen plays significant roles in many of the key reactions in the rumen system (Fig. 4). While glycolysis was, not surprisingly, a widely observed trait across several phylogenetic groups, mannose and fructose metabolism was mainly limited to strains of *P. ruminicola*, *F. succinogenes* and the uncultured UBA1213. *Prevotella ruminicola* and *F. succinogenes* additionally displayed a relatively high detection level of phosphotransacetylase (PF01515) related to acetate production, in addition to several of the key proteins related to the generation of propionate, mainly via oxaloacetate (Lactate/malate dehydrogenase [PF00056/PF02866], Methylmalonyl-CoA mutase [PF01642], Acetyl-CoA carboxylase [PF01039]) and fumarate (Succinate dehydogenase/fumarate reductase [PF12838], Fumarase [PF05681]). As expected due to the close phylogenetic relation to *Butyrivibrio*, genomic content of APb also revealed a metabolic capacity for butyrate production, and its active role in butyrate synthesis in the rumen was supported by the detection of these proteins (Acetyl-CoA acetyltransferase [PF00108], 3-hydroxyacyl-CoA dehydrogenase [PF00725/PF02737], Enoyl-CoA hydratase/isomerase [PF00378], Acyl-CoA dehydrogenase [PF00441]) in the metaproteome data (Fig. 4).

Although anaerobic fungi have been reported to participate in rumen fermentation, only a few genes related to for example acetate production seem to be “switched on” at the sampling timepoint for our dataset. This may be due to slow growth rates and low protein abundance for these gene sets. Furthermore, while complete glycolysis pathways are annotated for all currently cultivated fungal genomes, only a full set of glycolysis proteins aligning to the genes of *Neocallimastix* was detected at high protein detection levels in our metaproteome data, suggesting that anaerobic fungi only play a minor active role in the downstream carbon flow. It should also be noted that while the overall detection of proteins associated to the fungal population was higher in switchgrass fiber compared to rumen fluid, the opposite was observed for proteins associated to the abovementioned *Prevotella* clade (Supplementary Fig. S4). Seen in context with the high detection level of fungal enzymes for cellulose decomposition, this emphasizes that a key role of anaerobic fungi during this phase of the biomass degradation (48 h) is likely to function in recalcitrant fiber degradation of lignin-enriched fiber residues, whereas bacteria encompass a wider functional repertoire, including degradation of more easily digestible fibers and fermentation.

## Conclusions

While our understanding of the rumen microbiome has increased significantly in recent years, the majority of this knowledge has been restricted to the bacterial population. Insights into the role of anaerobic rumen fungi have been limited to a few studies and still very little is known about the overall ecology of anaerobic rumen fungi as part of the rumen microbiome and their contribution to the biomass-degrading process in the native habitat. In the current study, we report a time-dependent scenario within the rumen ecosystem where bacteria appear to have occupied multiple functional niches, while anaerobic fungi seem to dictate the degradation of resilient lignocellulosic plant material. Here, members of the GH family GH48 were detected at elevated levels and appeared to come nearly exclusively from the rumen fungi. Furthermore, it appears as if the bacterial population in the rumen is primarily involved in degradation of hemicellulose, at least for plant material that has been incubated in the rumen for 48 h. Overall, these results suggest that anaerobic fungi have a strongly adherent phenotype and colonize recalcitrant plant cell wall material that is likely too large in dimension/particle size to pass out of the rumen. Furthermore, we speculate that their adherent strategy is to maintain their population size in the rumen and prevent them from being washed out, given that they grow slower than the general rumen turnover rate. Although these results broaden our understanding of the native function of anaerobic rumen fungi, spatial and temporal experiments would certainly be beneficial to provide further support of the hypothesis that the detected proteins are ubiquitously involved in the degradation of recalcitrant biomass in the rumen and are essential to the nutrition and well-being of their host animal.

## Supplementary information

Supplementary material

Supplementary Data S1

Supplementary Data S2

Supplementary Data S3

## Data Availability

The mass spectrometry proteomics data have been deposited to the ProteomeXchange Consortium (http://proteomecentral.proteomexchange.org) via the PRIDE [76] partner repository with the dataset identifier PXD017007. The metatranscriptome raw files are submitted to NCBI SRA, accession numbers SRR9001933, SRR6230176, SRR6230410, SRR9001942, SRR9002087 and SRR6230409. The references for the genomes, metagenome-assembled genomes and viral scaffolds are listed in Supplementary Table S1. The databases used in this study, RUS-refDB and MT-eukDB, can be found in FigShare (https://figshare.com), DOI:10.6084/m9.figshare.12400577 and DOI:10.6084/m9.figshare.12385511, respectively. The viral scaffolds (nt) are also available via FigShare: DOI:10.6084/m9.figshare.12385505.
