# Supplementary material for: Proteome specialization of anaerobic fungi during ruminal degradation of recalcitrant plant fiber
Source: ISME J. 2020 Sep 14;15(2):421–34. doi: 10.1038/s41396-020-00769-x (PMC8026616; doi:10.1038/s41396-020-00769-x)
Supplement: Supplementary file 1 — Supplementary material [file 41396_2020_769_MOESM1_ESM.docx]

**SUPPLEMENTARY MATERIAL**

**Proteome specialization of anaerobic fungi during ruminal degradation of recalcitrant plant fiber**

^🖂^Live H. Hagen, Charles G. Brooke, Claire A. Shaw, Angela D. Norbeck, Hailan Piao, Magnus Ø. Arntzen, Heather M. Olson, Alex Copeland, Nancy Isern, Anil Shukla, Simon Roux, Vincent Lombard, Bernard Henrissat, Michelle A. O’Malley, Igor V. Grigoriev, Susannah G.Tringe, Roderick I. Mackie, Ljiljana Pasa-Tolic, Phillip B. Pope, and Matthias Hess

^🖂^Corresponding author:

Live H. Hagen – [live.hagen@nmbu.no](mailto:live.hagen@nmbu.no)

*List of content:*

**Figure S1:** Quantitative breakdown of **(A)** detected protein groups and **(B)** protein groups annotated as CAZyme when searching the metaproteome against the ‘RUS-refDB’ database.

**Figure S2:** Predicted CAZymes in the genetic content of the rumen virome.

**Figure S3:** CAZyme profile from each predicted source organism in RUS-refDB, displaying the detected proteins associated with both microhabitats (rumen fluid and switchgrass fiber).

**Figure S4:** Metabolic reconstruction of key players intermediate rumen fermentation as determined in both microhabitats (rumen fluid and switchgrass fiber).

**Figure S5:** Correlation analysis of the proteins quantified between the two biological replicates.

**Table S1:** Overview of the genomes, metagenome-assembled genomes (MAGs) and the viral scaffolds (MAVs) used to generate RUS-refDB.

**Table S2:** Table of detected protein groups associated with cellulosome signature domains.

**Table S3:** Metatranscriptome Quality Filtering Stats.

**Text S1:** Supplementary Results and Discussion.

**Text S2:** Supplementary Material and Methods.

**Text S3:** FASTA sequences of **(A)** viral protein sequences detected within the metaproteome and **(B)** proteins annotated as GH48 in MT-eukDB.

*In separate files:*

**Supplementary Data S1**: The complete concatenated ribosomal protein tree in Newick format.

**Supplementary Data S2:** Extended table of detected protein groups mapping against RUS-refDB.

**Supplementary Data S3:** Extended table of detected protein groups annotated as CAZymes mapping against MT-eukDB.

**Supplementary Figures and Tables**


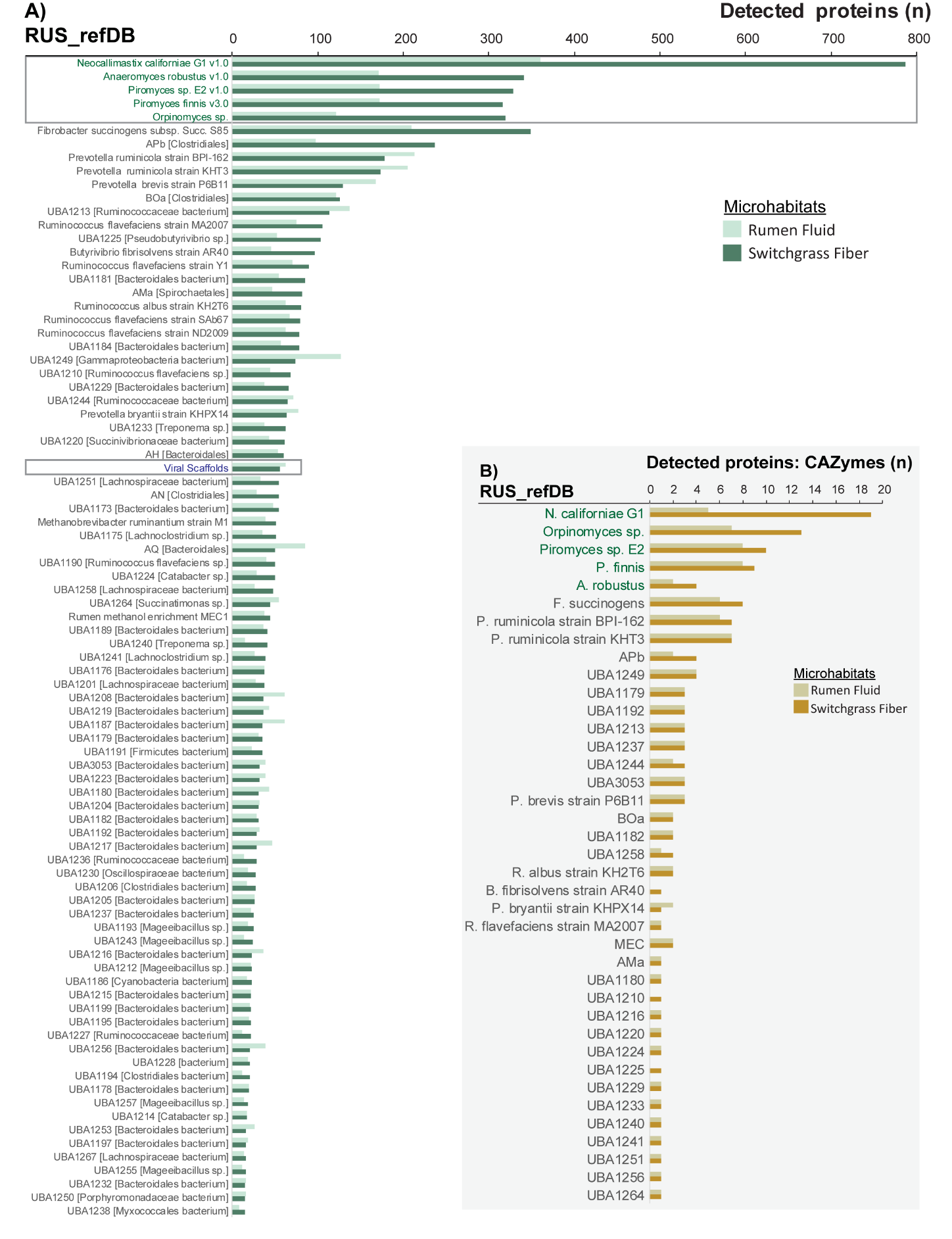


**Figure S1:** Quantitative breakdown of **(A)** detected protein groups (Genomes/MAGs/MAVs with > 15 protein groups detected are displayed) and **(B)** detected protein groups annotated as CAZymes (includes GHs, CEs, GTs, CBMs and dockerins) when searching the metaproteome against the ‘RUS-refDB’ database. Fungal genomes are highlighted with green font color, viral scaffolds in blue and prokaryotic genomes/MAGs are written in grey. Only proteins detected in both animals in at least one of the two microhabitats were included. Moreover, if a protein group consisted of more than one protein identification, only the first identification was considered.


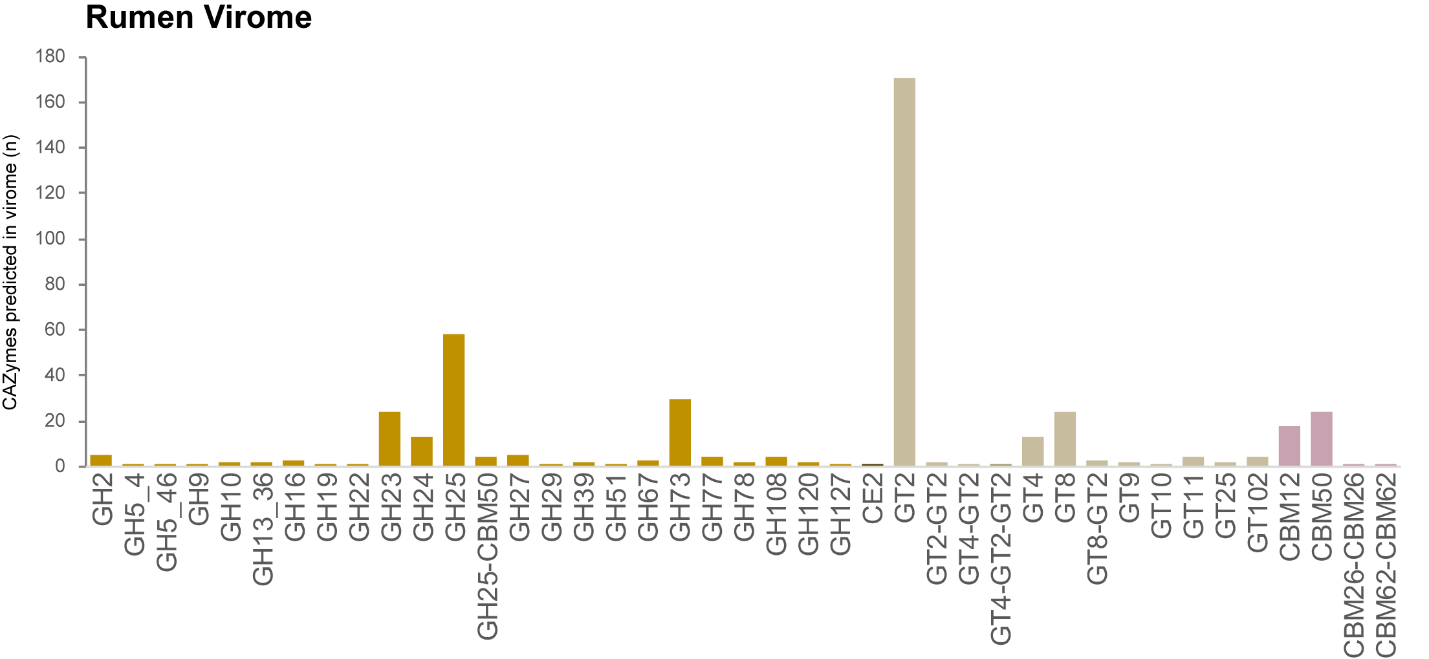


**Figure S2:** Predicted CAZymes in the genetic content of the rumen virome, colored by enzyme classes (GH, Glycoside Hydrolases; CE, Carbohydrate Esterases, GT, GlycosylTransferases; CBM; Carbohydrate-Binding Modules). None of these were detected in the metaproteomics data.


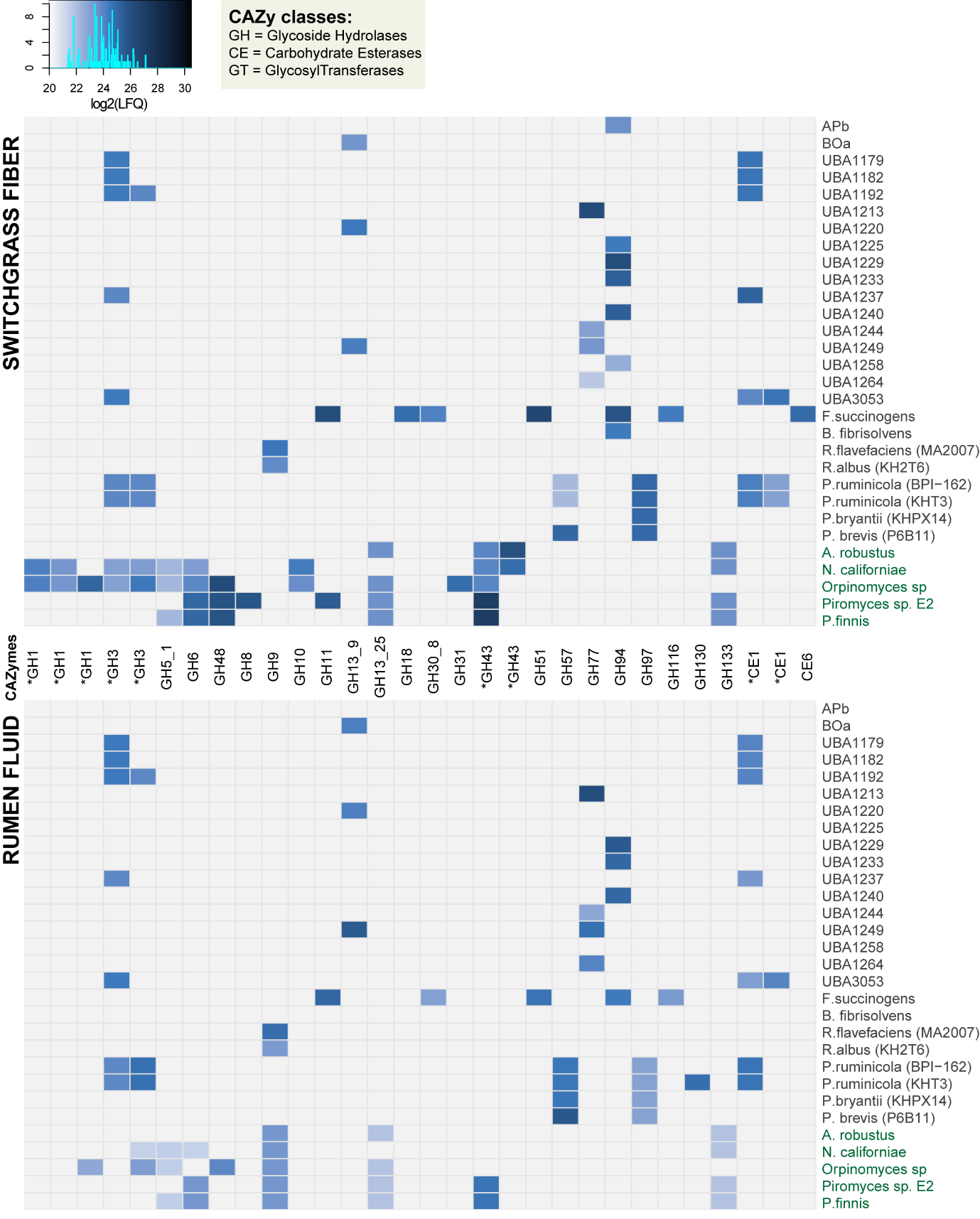


**Figure S3:** CAZyme profile from each predicted source organism in RUS-refDB, illustrating the detected proteins associated with the milled switchgrass fiber (top) and rumen fluid (bottom). Only CAZymes detected in both animals in at least one of the microhabitats are displayed to achieve high confidence detection of the active key populations. If more than one variant of a CAZyme family is detected, the log_2_(LFQ)-score is reported for each variant (indicated with *). The colors in the heat map indicate the protein detection levels of each protein group reported as the average Log_2_(LFQ)-scores for the biological replicates, where a light blue color is low detection while darker is high protein detection.


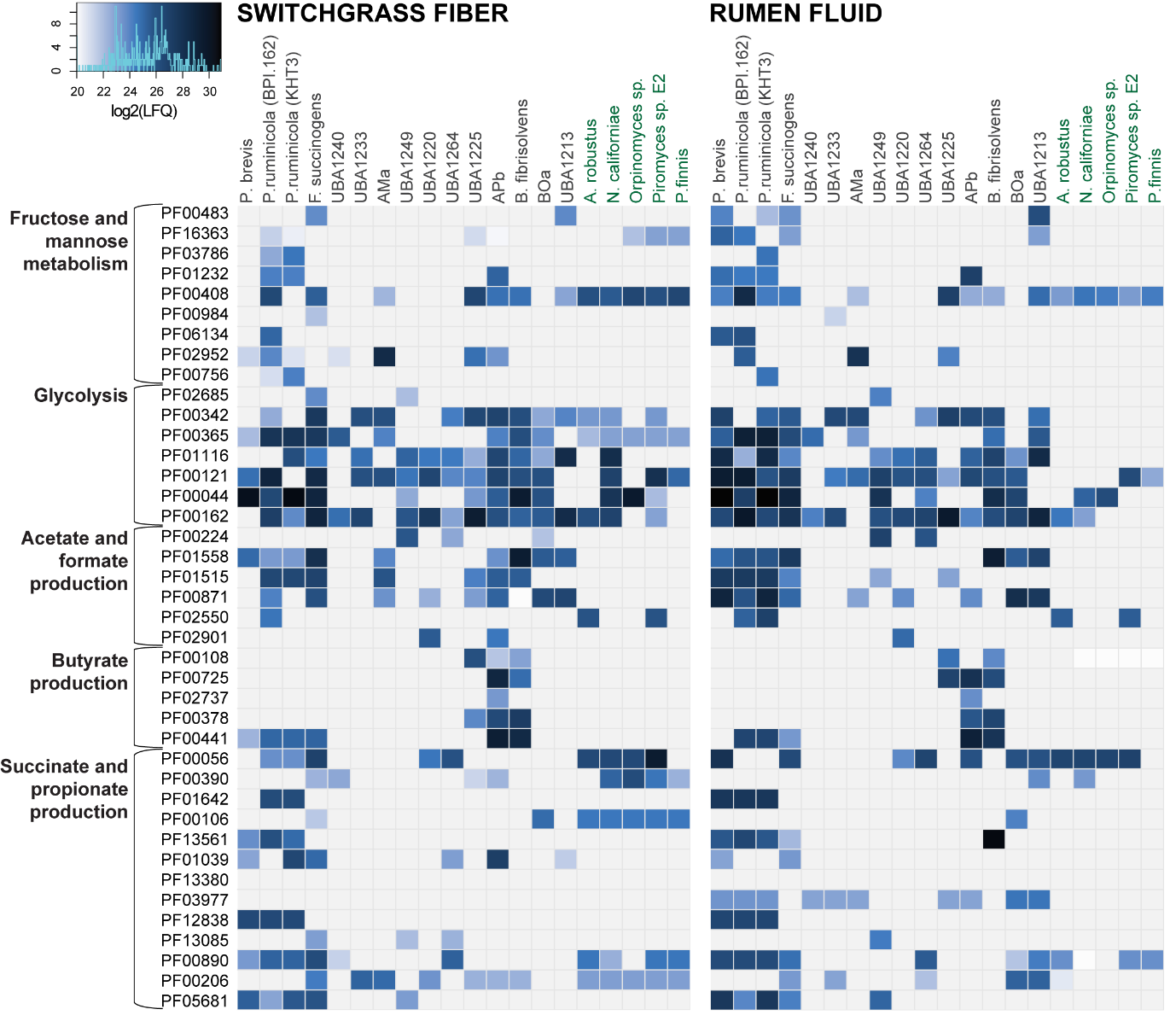


**Figure S4**: Metabolic reconstruction of key players intermediate rumen fermentation as determined in this study. The heat map shows the detection of proteins associated to main metabolic pathways (listed as pfam IDs) found in the most active genomes/MAGs in RUS-refDB. Detection of proteins associated with both switchgrass (left) and rumen fluid (right) are shown. The colors in the heat map indicate the protein detection levels reported as the average log_2_(LFQ)-scores for each biological replicate, where light blue represent lower detection levels while darker blue is high protein detection.


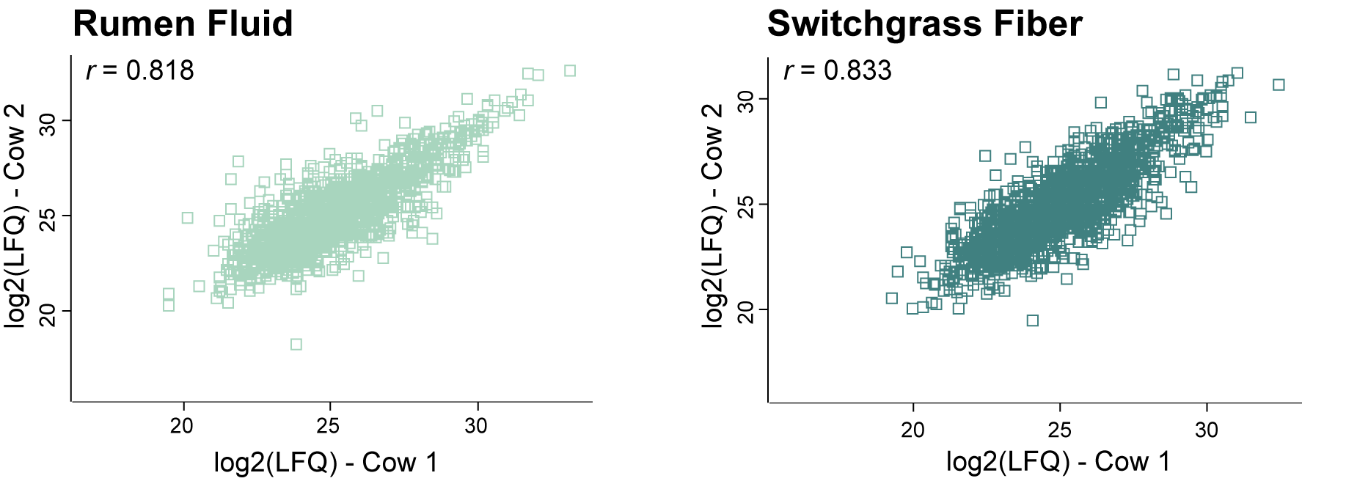


**Figure S5**: Correlation analysis of log_2_(LFQ) values between the two biological replicates (Cow 1 and Cow 2) in the rumen fluid and switchgrass fiber microhabitats. The Pearson coefficient for each comparison is shown in each plot.

**Table S1**: Overview of the genomes, metagenome-assembled genomes (MAGs) and the virome used to generate RUS-refDB. This table includes the number of predicted genes, as well as the number of detected proteins in the rumen fluid (RF) and switchgsrass fiber (SF) when mapped against the metaproteomics data.

| **IDs in MP** | **Organism Name** | **Ref.** | **Predicted Genes (n)** | **Detected proteins (n)** | | |
| --- | --- | --- | --- | --- | --- | --- |
|  |  |  |  | ***RF*** | ***SF*** | |
| **Genomes of anaerobic cultivated fungi** | | | | | | |
| jgi\|Anasp1 | Anaeromyces robustus v1.0 | [4] | 12832 | 171 | | 341 |
| jgi\|Neosp1 | Neocallimastix californiae G1 v1.0 | [4] | 20219 | 360 | | 787 |
| jgi\|Orpsp1_1 | Orpinomyces sp. | [18] | 18936 | 121 | | 320 |
| jgi\|PirE2_1 | Piromyces sp. E2 v1.0 | [4] | 14648 | 172 | | 329 |
| jgi\|Pirfi3 | Piromyces finnis v3.0 | [4] | 10992 | 172 | | 316 |
| **MAGs from a rumen metagenome** | | | | | | |
| AH | AH [Bacteroidales] | [31] | 3652 | 53 | | 60 |
| AMa | AMa [Spirochaetales] | [31] | 3103 | 47 | | 82 |
| AN | AN [Clostridiales] | [31] | 3967 | 29 | | 55 |
| APb | APb [Clostridiales] | [31] | 4239 | 97 | | 237 |
| AQ | AQ [Bacteroidales] | [31] | 3151 | 85 | | 50 |
| AS1a | AS1a [Clostridiales] | [31] | 2302 | 15 | | 11 |
| BOa | BOa [Clostridiales] | [31] | 4188 | 121 | | 126 |
| UBA3053 | UBA3053 [Bacteroidales bacterium] | [30] | 1694 | 39 | | 32 |
| UBA3054 | UBA3054 [Candidatus UBP3 bacterium] | [30] | 1217 | 4 | | 3 |
| UBA1173 | UBA1173 [Bacteroidales bacterium] | [30] | 1992 | 48 | | 54 |
| UBA1174 | UBA1174 [Elusimicrobia bacterium] | [30] | 1248 | 4 | | 5 |
| UBA1175 | UBA1175 [Lachnoclostridium sp.] | [30] | 2287 | 35 | | 51 |
| UBA1176 | UBA1176 [Bacteroidales bacterium] | [30] | 2144 | 37 | | 38 |
| UBA1177 | UBA1177 [Candidatus UBP6 bacterium] | [30] | 1931 | 3 | | 5 |
| UBA1178 | UBA1178 [Bacteroidales bacterium] | [30] | 1410 | 19 | | 20 |
| UBA1179 | UBA1179 [Bacteroidales bacterium] | [30] | 1699 | 31 | | 35 |
| UBA1180 | UBA1180 [Bacteroidales bacterium] | [30] | 1629 | 43 | | 31 |
| UBA1181 | UBA1181 [Bacteroidales bacterium] | [30] | 2604 | 55 | | 85 |
| UBA1182 | UBA1182 [Bacteroidales bacterium] | [30] | 2090 | 28 | | 31 |
| UBA1183 | UBA1183 [Verrucomicrobia bacterium] | [30] | 1000 | 1 | | 0 |
| UBA1184 | UBA1184 [Bacteroidales bacterium] | [30] | 2301 | 57 | | 78 |
| UBA1185 | UBA1185 [Alphaproteobacteria bacterium] | [30] | 792 | 3 | | 0 |
| UBA1186 | UBA1186 [Cyanobacteria bacterium] | [30] | 1625 | 17 | | 23 |
| UBA1187 | UBA1187 [Bacteroidales bacterium] | [30] | 1477 | 61 | | 35 |
| UBA1188 | UBA1188 [Bacillales bacterium] | [30] | 1004 | 7 | | 6 |
| UBA1189 | UBA1189 [Bacteroidales bacterium] | [30] | 2498 | 36 | | 41 |
| UBA1190 | UBA1190 [Ruminococcus flavefaciens sp.] | [30] | 2946 | 40 | | 50 |
| UBA1191 | UBA1191 [Firmicutes bacterium] | [30] | 1316 | 23 | | 35 |
| UBA1192 | UBA1192 [Bacteroidales bacterium] | [30] | 1833 | 32 | | 29 |
| UBA1193 | UBA1193 [Mageeibacillus sp.] | [30] | 2111 | 18 | | 25 |
| UBA1194 | UBA1194 [Clostridiales bacterium] | [30] | 1990 | 12 | | 21 |
| UBA1195 | UBA1195 [Bacteroidales bacterium] | [30] | 1997 | 20 | | 22 |
| UBA1196 | UBA1196 [Candidatus UBP6 bacterium] | [30] | 1693 | 3 | | 4 |
| UBA1197 | UBA1197 [Bacteroidales bacterium] | [30] | 1802 | 18 | | 16 |
| UBA1198 | UBA1198 [Faecalibacterium sp.] | [30] | 1299 | 8 | | 10 |
| UBA1199 | UBA1199 [Bacteroidales bacterium] | [30] | 1191 | 21 | | 22 |
| UBA1200 | UBA1200 [Verrucomicrobia bacterium] | [30] | 2626 | 9 | | 11 |
| UBA1201 | UBA1201 [Lachnospiraceae bacterium] | [30] | 2105 | 27 | | 37 |
| UBA1202 | UBA1202 [Elusimicrobia bacterium] | [30] | 1288 | 5 | | 6 |
| UBA1203 | UBA1203 [Myxococcales bacterium] | [30] | 2161 | 0 | | 12 |
| UBA1204 | UBA1204 [Bacteroidales bacterium] | [30] | 2202 | 32 | | 31 |
| UBA1205 | UBA1205 [Bacteroidales bacterium] | [30] | 2054 | 26 | | 26 |
| UBA1206 | UBA1206 [Clostridiales bacterium] | [30] | 1747 | 17 | | 27 |
| UBA1207 | UBA1207 [Ruminococcaceae bacterium] | [30] | 1622 | 13 | | 14 |
| UBA1208 | UBA1208 [Bacteroidales bacterium] | [30] | 2405 | 61 | | 36 |
| UBA1209 | UBA1209 [Candidatus UBP6 bacterium] | [30] | 1647 | 5 | | 8 |
| UBA1210 | UBA1210 [Ruminococcus flavefaciens sp.] | [30] | 2272 | 44 | | 68 |
| UBA1211 | UBA1211 [Verrucomicrobia bacterium] | [30] | 2824 | 10 | | 12 |
| UBA1212 | UBA1212 [Mageeibacillus sp.] | [30] | 2189 | 22 | | 23 |
| UBA1213 | UBA1213 [Ruminococcaceae bacterium] | [30] | 2202 | 137 | | 113 |
| UBA1214 | UBA1214 [Catabacter sp.] | [30] | 1758 | 17 | | 17 |
| UBA1215 | UBA1215 [Bacteroidales bacterium] | [30] | 1559 | 22 | | 22 |
| UBA1216 | UBA1216 [Bacteroidales bacterium] | [30] | 1810 | 36 | | 23 |
| UBA1217 | UBA1217 [Bacteroidales bacterium] | [30] | 1792 | 47 | | 28 |
| UBA1218 | UBA1218 [Alphaproteobacteria bacterium] | [30] | 812 | 1 | | 3 |
| UBA1219 | UBA1219 [Bacteroidales bacterium] | [30] | 2281 | 43 | | 36 |
| UBA1220 | UBA1220 [Succinivibrionaceae bacterium] | [30] | 1140 | 43 | | 61 |
| UBA1221 | UBA1221 [Cyanobacteria bacterium] | [30] | 2139 | 11 | | 11 |
| UBA1222 | UBA1222 [Dehalococcoidia bacterium] | [30] | 815 | 5 | | 5 |
| UBA1223 | UBA1223 [Bacteroidales bacterium] | [30] | 1650 | 39 | | 32 |
| UBA1224 | UBA1224 [Catabacter sp.] | [30] | 2733 | 28 | | 50 |
| UBA1225 | UBA1225 [Pseudobutyrivibrio sp.] | [30] | 2646 | 52 | | 103 |
| UBA1227 | UBA1227 [Ruminococcaceae bacterium] | [30] | 977 | 11 | | 22 |
| UBA1228 | UBA1228 [bacterium] | [30] | 1943 | 18 | | 21 |
| UBA1229 | UBA1229 [Bacteroidales bacterium] | [30] | 2972 | 37 | | 66 |
| UBA1230 | UBA1230 [Oscillospiraceae bacterium] | [30] | 1402 | 18 | | 27 |
| UBA1231 | UBA1231 [Bacillales bacterium] | [30] | 1279 | 7 | | 10 |
| UBA1232 | UBA1232 [Bacteroidales bacterium] | [30] | 1771 | 16 | | 15 |
| UBA1233 | UBA1233 [Treponema sp.] | [30] | 2880 | 37 | | 62 |
| UBA1234 | UBA1234 [Clostridiales bacterium] | [30] | 1456 | 4 | | 9 |
| UBA1235 | UBA1235 [Ca. Saccharibacteria bacterium] | [30] | 603 | 0 | | 0 |
| UBA1236 | UBA1236 [Ruminococcaceae bacterium] | [30] | 1514 | 14 | | 28 |
| UBA1237 | UBA1237 [Bacteroidales bacterium] | [30] | 2153 | 22 | | 25 |
| UBA1238 | UBA1238 [Myxococcales bacterium] | [30] | 3136 | 8 | | 15 |
| UBA1239 | UBA1239 [Clostridiales bacterium] | [30] | 1354 | 5 | | 10 |
| UBA1240 | UBA1240 [Treponema sp.] | [30] | 1972 | 15 | | 41 |
| UBA1241 | UBA1241 [Lachnoclostridium sp.] | [30] | 2012 | 26 | | 39 |
| UBA1242 | UBA1242 [Catabacter sp.] | [30] | 1070 | 3 | | 8 |
| UBA1243 | UBA1243 [Mageeibacillus sp.] | [30] | 1518 | 14 | | 24 |
| UBA1244 | UBA1244 [Ruminococcaceae bacterium] | [30] | 2075 | 71 | | 65 |
| UBA1245 | UBA1245 [Bacillales bacterium] | [30] | 628 | 0 | | 4 |
| UBA1246 | UBA1246 [Clostridiales bacterium] | [30] | 1884 | 11 | | 12 |
| UBA1247 | UBA1247 [Candidatus UBP3 bacterium] | [30] | 1250 | 2 | | 3 |
| UBA1248 | UBA1248 [Clostridiales bacterium] | [30] | 1983 | 9 | | 14 |
| UBA1249 | UBA1249 [Gammaproteobacteria bacterium] | [30] | 1377 | 127 | | 74 |
| UBA1250 | UBA1250 [Porphyromonadaceae bacterium] | [30] | 1614 | 16 | | 15 |
| UBA1251 | UBA1251 [Lachnospiraceae bacterium] | [30] | 1785 | 33 | | 55 |
| UBA1252 | UBA1252 [Catabacter sp.] | [30] | 1212 | 6 | | 13 |
| UBA1253 | UBA1253 [Bacteroidales bacterium] | [30] | 1677 | 26 | | 16 |
| UBA1254 | UBA1254 [Alphaproteobacteria bacterium] | [30] | 851 | 4 | | 0 |
| UBA1255 | UBA1255 [Mageeibacillus sp.] | [30] | 2126 | 12 | | 16 |
| UBA1256 | UBA1256 [Bacteroidales bacterium] | [30] | 1352 | 39 | | 21 |
| UBA1257 | UBA1257 [Mageeibacillus sp.] | [30] | 1446 | 14 | | 18 |
| UBA1258 | UBA1258 [Lachnospiraceae bacterium] | [30] | 2042 | 26 | | 48 |
| UBA1259 | UBA1259 [Catabacter sp.] | [30] | 1179 | 9 | | 12 |
| UBA1260 | UBA1260 [Catabacter sp.] | [30] | 1179 | 12 | | 11 |
| UBA1261 | UBA1261 [Bacillales bacterium] | [30] | 1194 | 4 | | 5 |
| UBA1262 | UBA1262 [Catabacter sp.] | [30] | 716 | 12 | | 8 |
| UBA1263 | UBA1263 [Verrucomicrobia bacterium] | [30] | 1911 | 9 | | 8 |
| UBA1264 | UBA1264 [Succinatimonas sp.] | [30] | 1152 | 54 | | 44 |
| UBA1265 | UBA1265 [Spirochaetales bacterium] | [30] | 1377 | 9 | | 12 |
| UBA1266 | UBA1266 [Bacteroidales bacterium] | [30] | 1557 | 10 | | 8 |
| UBA1267 | UBA1267 [Lachnospiraceae bacterium] | [30] | 1424 | 14 | | 16 |
| **Reference bacterial/archaeal genomes** | | | | | | |
| Ga0070636 | Prevotella ruminicola strain BPI-162 | [6] | 3012 | 213 | | 178 |
| Ga0104360 | P. ruminicola strain KHT3 | [6] | 3446 | 205 | | 173 |
| T496DRAFT | P. brevis strain P6B11 | [6] | 2510 | 168 | | 129 |
| Ga0104364 | P. bryantii strain KHPX14 | [6] | 2707 | 77 | | 64 |
| Ga0104370 | Ruminococcus albus strain KH2T6 | [6] | 3513 | 62 | | 81 |
| Ga0066885 | R. flavefaciens strain Y1 | [6] | 3199 | 70 | | 90 |
| T497DRAFT | R. flavefaciens strain MA2007 | [6] | 2907 | 75 | | 106 |
| T488DRAFT | R. flavefaciens strain ND2009 | [6] | 3137 | 63 | | 78 |
| IE37DRAFT | R. flavefaciens strain SAb67 | [6] | 3384 | 67 | | 79 |
| Ga0070258 | Bacillus licheniformis strain VTM3R78 | [6] | 4158 | 12 | | 9 |
| Ga0070254 | Butyrivibrio fibrisolvens strain AR40 | [6] | 4211 | 46 | | 96 |
| WP_012819846.1 | Fibrobacter succinogens subsp. Succ. S85 | [32] | 3188 | 210 | | 349 |
| WP_012954809.1 | Methanobrevibacter ruminantium strain M1 | [33] | 2135 | 39 | | 51 |
| Ga0117888_1000011 | Rumen methanol enrichment MEC1 | [6] | 44933 | 38 | | 44 |
| **Viral content from a rumen metagenome** | | | | | | |
| Vir | ViralScaffolds-RumenMG | [31,55] | 74440 | 62 | | 56 |

**Table S2**: Extended table of detected protein groups associated with cellulosome signature domains (dockerins). Protein detection level is given as log_2_(LFQ) for each biological replicate in samples from rumen fluid (RF) and switchgrass fiber (SF). Only the first protein identification per protein group is shown in this table. Protein groups with more than one protein sequence identification are indicated with *, and a complete list of protein IDs can be found in **Supplementary Data S2**. CAZyme modules detected at high confidence level (i.e. detected in both animals in at least one microhabitat) is indicated in bold font.

| **MAG/genome**  **[majority protein ID]** | **CAZyme module** | **Protein detection [log_2_(LFQ)]** | | | |
| --- | --- | --- | --- | --- | --- |
|  | *Microhabitat:*  *Cow:* | **RF 1** | **RF 2** | **SF**  **1** | **SF**  **2** |
| R. flavefaciens strain MA2007 [T497DRAFT_00845]* | **CBM4-GH9-DOC1** | 25.1 | 24.6 | 24.1 | 25.0 |
| R. flavefaciens strain MA2007 [T497DRAFT_00649] | DOC1 | 23.3 | NaN | NaN | NaN |
| R. flavefaciens strain MA2007 [T497DRAFT_01962]* | DOC1 | NaN | NaN | NaN | 20.6 |
| R. flavefaciens strain MA2007 [T497DRAFT_01936]* | GH11  GH11-CBM22-DOC1-CE1  GH11-CE1-DOC1-CE4  GH11-GH10  GH11-GH11-GH10-DOC1-GH11-CE4 | NaN | NaN | NaN | 22.8 |
| R. flavefaciens strain MA2007 [T497DRAFT_01436] | GH11-CBM22-GH10-DOC1-GH11-CE4 | NaN | 22.0 | NaN | NaN |
| R. flavefaciens strain MA2007 [T497DRAFT_00844] | GH9-CBM3-DOC1 | NaN | NaN | NaN | 21.3 |
| R. flavefaciens strain Y1 [Ga0066885_11723] | DOC1 | NaN | 23.8 | NaN | 23.3 |
| R. flavefaciens strain Y1 [Ga0066885_10689]* | GH11-CBM22-GH10-DOC1-CBM22-CE1  GH11-CBM22-GH10-DOC1-GH11-CE4 | NaN | NaN | NaN | 22.5 |
| UBA1190 [UBA1190_contig_427_14] | DOC1 | 22.9 | NaN | NaN | NaN |
| UBA1190 [UBA1190_contig_96797_16] | DOC1 | NaN | NaN | NaN | 19.4 |
| UBA1210 [UBA1210_contig_2415_7] | DOC1 | NaN | NaN | 21.4 | NaN |
| UBA1210 [UBA1210_contig_2415_8] | DOC1 | NaN | NaN | 24.7 | 23.9 |
| UBA1210 [UBA1210_contig_56792_10]* | GH11-CBM22-GH10-DOC1  GH11-CBM22-GH10-DOC1-CE1 | NaN | NaN | NaN | 22.7 |
| Anaeromyces robustus v1.0 [jgi\|Anasp1\|296357]* | CBM35-GH26-DOC2-DOC2  CBM35-GH26-DOC2-DOC2-DOC2-DOC2-DOC2 | NaN | NaN | NaN | 20.8 |
| Anaeromyces robustus v1.0 [jgi\|Anasp1\|327659]* | CE15  CE15-DOC2-DOC2  GH11-CBM6-CE15 | NaN | NaN | NaN | 24.4 |
| Anaeromyces robustus v1.0 [jgi\|Anasp1\|294808]* | GH3-DOC2-DOC2-DOC2 | NaN | NaN | 23.5 | NaN |
| Anaeromyces robustus v1.0 [jgi\|Anasp1\|287068]* | **GH43**  **GH43-CBM6**  **GH43-CBM6-CBM13-DOC2-DOC2**  **GH43-CBM6-DOC2-DOC2** | NaN | NaN | 23.9 | 24.0 |
| Anaeromyces robustus v1.0 [jgi\|Anasp1\|327938] | GH43-CBM6-DOC2-DOC2 | NaN | 20.8 | NaN | NaN |
| Anaeromyces robustus v1.0 [jgi\|Anasp1\|233234]* | GH48-DOC2-DOC2 | NaN | NaN | NaN | 26.0 |
| Anaeromyces robustus v1.0 [jgi\|Anasp1\|325830] | GH48-DOC2-DOC2 | NaN | NaN | NaN | 22.7 |
| Anaeromyces robustus v1.0 [jgi\|Anasp1\|293207]* | GH9-DOC2-DOC2  GH9-DOC2-DOC2-DOC2 | NaN | NaN | NaN | 21.6 |
| Piromyces finnis v3.0 [jgi\|Pirfi3\|344383]* | DOC2-DOC2-GH6  GH6 | NaN | NaN | 23.3 | NaN |
| Piromyces finnis v3.0 [jgi\|Pirfi3\|354732]* | **DOC2-DOC2-GH6**  **GH6** | 24.6 | 22.1 | 25.7 | 24.5 |
| Piromyces finnis v3.0 [jgi\|Pirfi3\|413312]* | GH48-DOC2-DOC2 | NaN | NaN | 23.4 | NaN |
| Piromyces sp. E2 v1.0 [jgi\|PirE2_1\|23096]* | CE3-GH11-CE3  GH11  GH11-GH11  GH11-GH11-DOC2  GH11-GH11-GH11 | NaN | NaN | NaN | 22.4 |
| Piromyces sp. E2 v1.0 [jgi\|PirE2_1\|47957]* | DOC2-DOC2-GH43  GH43 | NaN | NaN | NaN | 23.5 |
| Piromyces sp. E2 v1.0 [jgi\|PirE2_1\|11019]* | GH124-DOC2  GH124-DOC2-DOC2 | NaN | NaN | NaN | 22.0 |
| Piromyces sp. E2 v1.0 [jgi\|PirE2_1\|12703]* | **GH48-DOC2**  **GH48-DOC2-DOC2** | NaN | NaN | 25.7 | 25.9 |
| Piromyces sp. E2 v1.0 [jgi\|PirE2_1\|21620] | **GH8-DOC2-DOC2** | NaN | NaN | 25.7 | 25.8 |
| Piromyces sp. E2 v1.0 [jgi\|PirE2_1\|20181]* | GH9-DOC2-DOC2-DOC2 | NaN | NaN | NaN | 24.1 |
| Neocallimastix californiae G1 v1.0 [jgi\|Neosp1\|699681]* | CBM13-DOC2-DOC2-CE1  CE1  DOC2-CE1  DOC2-DOC2-CE1 | NaN | NaN | 23.6 | NaN |
| Neocallimastix californiae G1 v1.0 [jgi\|Neosp1\|177128]* | **DOC2-DOC2-DOC2-GH5_1**  **DOC2-GH5_1GH5_1** | 21.5 | NaN | 22.4 | 22.0 |
| Neocallimastix californiae G1 v1.0 [jgi\|Neosp1\|514294]* | DOC2-DOC2-GH5_4-GH5_4-GH5_4-DOC2-DOC2  GH5_4-GH5_4-DOC2-DOC2 | NaN | 22.1 | NaN | NaN |
| Neocallimastix californiae G1 v1.0 [jgi\|Neosp1\|708206]* | GH2  GH2-CBM13  GH2-CBM13-DOC2  GH2-CBM13-DOC2-DOC2 | NaN | NaN | 23.3 | NaN |
| Neocallimastix californiae G1 v1.0 [jgi\|Neosp1\|699432] | GH3-DOC2-GH6-DOC2-DOC2 | NaN | 21.4 | 23.1 | NaN |
| Neocallimastix californiae G1 v1.0 [jgi\|Neosp1\|503798] | GH45-DOC2-DOC2-DOC2 | NaN | NaN | NaN | 20.8 |
| Neocallimastix californiae G1 v1.0 [jgi\|Neosp1\|704451] | GH48-DOC2-DOC2 | NaN | NaN | 24.4 | NaN |
| Neocallimastix californiae G1 v1.0 [jgi\|Neosp1\|705896] | GH48-DOC2-DOC2 | NaN | NaN | 23.7 | NaN |
| Neocallimastix californiae G1 v1.0 [jgi\|Neosp1\|675650]* | GH8-DOC2  GH8-DOC2-DOC2 | NaN | 25.0 | NaN | NaN |
| Orpinomyces sp. [jgi\|Orpsp1_1\|1178620]* | DOC2-DOC2 | NaN | NaN | NaN | 22.2 |
| Orpinomyces sp. [jgi\|Orpsp1_1\|1188096]* | DOC2-DOC2 | NaN | NaN | 22.5 | NaN |
| Orpinomyces sp. [jgi\|Orpsp1_1\|1188535] | DOC2-DOC2 | NaN | NaN | NaN | 25.4 |
| Orpinomyces sp. [jgi\|Orpsp1_1\|1176933]* | DOC2-DOC2-DOC2-GH6 | NaN | NaN | NaN | 23.1 |
| Orpinomyces sp. [jgi\|Orpsp1_1\|1179777]* | **DOC2-DOC2-DOC2-GH9**  **GH9** | 23.7 | 23.0 | NaN | NaN |
| Orpinomyces sp. [jgi\|Orpsp1_1\|1175496] | **DOC2-DOC2-GH6** | NaN | NaN | 24.2 | 23.6 |
| Orpinomyces sp. [jgi\|Orpsp1_1\|1178177]* | DOC2-DOC2-GH6  DOC2-GH6 | NaN | NaN | 21.6 | NaN |
| Orpinomyces sp. [jgi\|Orpsp1_1\|1176624]* | DOC2-DOC2-GH6  DOC2-GH6;GH6 | NaN | NaN | NaN | 20.9 |
| Orpinomyces sp. [jgi\|Orpsp1_1\|1176522]* | GH3-DOC2-GH6-DOC2-DOC2 | NaN | NaN | NaN | 23.3 |
| Orpinomyces sp. [jgi\|Orpsp1_1\|1182666]* | GH43  GH43-CBM6-CBM13  GH43-CBM6-CBM13-DOC2  GH43-CBM6-CBM13-DOC2-DOC2  GH43-CBM6-DOC2  GH43-CBM6-DOC2-DOC2 | NaN | NaN | NaN | 24.6 |
| Orpinomyces sp. [jgi\|Orpsp1_1\|1182381] | **GH48-DOC2-DOC2** | NaN | 23.9 | 26.6 | 25.9 |

**Table S3:** Metatranscriptome Quality Filtering Stats.

|  | **Total reads**  **[count(%)]** | **Low quality reads [count(%)]** | **Artifact reads [count(%)]** | **rRNA reads [count(%)]** | **Remaining reads [count(%)]** |
| --- | --- | --- | --- | --- | --- |
| **Rumen Metatranscriptome** | 423 409 432  (100) | 3 000 746  (4.78) | 119 751 (0.15) | 4 245 680 (5.26) | 379 212 367  (89.56) |

**Supplementary Text**

**Text S1: Results and Discussion**

**Virome activity in ruminant biomass-degradation**

To further enhance our understanding of the role of rumen viruses and how they might shape the different microbial populations within the rumen ecosystem, we analyzed the proteins that were detected in our metaproteomes and that originated from genomic material of viral origin. In accordance with recent research efforts to elucidate the role of the rumen virome, a significant portion of the proteins (switchgrass: 56; rumen fluid: 62) were assigned to the 913 viral scaffolds we recovered from our switchgrass-associated rumen metagenome^1,2^ (**Supplementary Figure S1**). Recent studies have shed light on the viral rumen population and although work in this area is still nascent, it suggests that the rumen virome modulates carbon cycling within the rumen ecosystem directly, as they encode glycoside hydrolases^3^, or indirectly through cell lysis or re-programming of the metabolism of the host microbiome^4–6^. Accordingly, when mining the genomic content of the viral scaffolds, we identified CAZyme domains within 444 protein-coding genes (**Supplementary Figure S2**). The two most prominent was glycosyltransferases (family 2; 171 genes) and glycoside hydrolase family 25 (58 genes). Glycosyltransferases, either exploited from the host or expressed independently by the virus, are typically utilized for viral glycosylation to protect their DNA from host restriction endonucleases or to modify the serotype of the host^7^. GH25 contains dominantly enzymes that can hydrolyze the β-1,4-glycosidic bond between N-acetylmuramic acid and N- acetylglucosamine in the carbohydrate backbone of bacterial peptidoglycan and are essential to modify and lyse the bacterial cell wall^8,9^ contributing to intra-ruminal nitrogen turnover. Nevertheless, none of the predicted viral CAZymes were amongst the detected proteins. In general, only a few putative auxiliary metabolic genes were detected within metaproteomes; three bacterial extracellular solute-binding proteins and an oxidoreductase, consistent with a potentially indirect role of viruses in supporting biomass degradation (protein sequences in **Supplementary Text S3)**. Also two ribosomal proteins were found amongst the detected proteins in our data, further reinforcing a recent observation indicating that viruses can modulate the translation upon infection as a strategy to exploit its host^10^. Not surprisingly, a vast majority of the detected viral proteins could not be assigned to any known function, and their purpose in the microbiome cannot be assessed at this time and will require further protein characterization efforts. Several of the detected viral-associated protein groups showed low redundancy and relatively high protein abundance, including a protein detected at the upper range of the protein detection level (average log_2_(LFQ) score = 31.5; gene ID ‘Vir_gene_id_42007’ in **Supplementary Data 2,** the protein sequence can be found in **Supplementary Text S3**). This protein showed high homology (using Phyre^2^: Protein Homology/AnalogY Recognition Engine) to a porter protein, directly involved in the capsid formation and previously found highly abundant in a virion-associated metaproteome^11^. Notably, this protein was detected in the switchgrass fiber fraction samples, yet was absent in the rumen fluid samples. Overall, the numerous viral proteins observed in this study, several quantified at high protein detection level demonstrating their presence and activity, strongly advocate the need for comprehensively studying the rumen virome.

**High prevalence of multi-modular domains and cellulosomal proteins**

Some of the most efficient biomass degrading anaerobes possess cellulosomes, which are multienzyme complexes that enable the orchestrated and synchronized activity of various enzymes that are needed to degrade the cellulosic and hemicellulosic components of recalcitrant plant material^12^. Until recently, cellulosomes and their essential building blocks have been identified and described only from anaerobic bacteria^13–16^. However, advances in the isolation and cultivation of anaerobic fungi coupled with genome and transcriptome analyses have confirmed the presence of cellulosomes in anaerobic fungi for the well-synchronized deconstruction of plant carbohydrates^17^. Many CAZymes appear in multidomain modules, often comprising substrate-binding domains in addition to one or several domains specific for multifunctional GH families. Within our ruminal metaproteome, we detected proteins containing cellulosomal domains such as bacterial and fungal dockerins and carbohydrate-binding modules, which are specific for these large, multiprotein structures. These non-catalytic domains have recently been demonstrated to be numerous in anaerobic fungi, with an average of more than 300 non-catalytic dockerin domains encoded in the genome of each strain^17^. Accordingly, a significant number of the detected CAZymes in our metaproteome data contained at least one dockerin domain, with a clear preponderance of dockerins of fungal origin (**Supplementary Table S2**). In general, while the bacterial ecellulosome signature sequences encompassed a single Type-I dockerin (DOC1), the fungal counterparts frequently occurred as double or triple dockerins domains (here classified as type-II; DOC2). Dockerin domains in tandem repeats are indeed associated with fungal cellulosomes, and it is believed that this construction facilitates the involvement of more binding sites, thus binding potential substrates more efficiently, than single dockerins^17,18^.

The CAZymes containing dockerin domains in tandem repeats were further flanked with a variety of glycoside hydrolase domains, including those belonging to the GH3, GH5_1, GH6, GH8, GH9, GH43 and GH48 family. Notably, while GH3 and GH6 have recently been confirmed in fungal cellulosomes^17^, they seem to be absent in bacterial counterparts. Moreover, the GH48 enzymes detected in our metaproteome, except those affiliated with *Piromyces finnis*, contained two copies of dockerin domains (**Supplementary Table S2,** also see **Supplementary Data S2** for a complete list of relevant protein IDs), strongly suggesting that anaerobic fungi employ GH48 in multi-modular enzymatic complexes to efficiently degrade crystalline cellulose. This observation is consistent with the powerful degradation activity of fungal multi-modular complexes previously demonstrated by Haitjema *et al*.,^17^. Although fungal and bacterial dockerins are evolutionary divergent, members of bacterial GH48s have indeed been recognized as the main catalytic component of a processive cellulase in *Clostridium thermocellum* (i.e. *CelS*), exhibiting exo-cellulolytic activity^19^. Albeit at lower protein abundance, peptides also matched fungal cellulosome signature sequences containing carbohydrate binding modules (CBM6 and CBM13) together with GH43s and domains indicative for dockerins (**Supplementary Table S2**). CBM10, thought to be linked to fungal cellulosomes^18,20,21^, were not detected. Furthermore, in resemblance to the overall metaproteome landscape investigated in this study, the fungal CAZymes had high redundancy across the fungal species as well as high prevalence within each genome.

**Glycoside hydrolases amongst MAGs**

While our metaproteome data confirmed the enzymatic machineries of the previously mentioned characterized bacteria, proteins associated with recalcitrant cellulose decomposition were not restricted to these. The MAG of the uncultivated UBA1213, classified as a member of *Ruminococcaceae,* was associated with multi-domain proteins containing GH77 and GT35 at high abundance, whereas a close relative of UBA1213, ‘BOa’, mapped to multi-domain CAZymes possessing an α-amylase domain (i.e. GH13_9) and the carbohydrate binding module CBM48. Both these modules have been shown to be involved in starch degradation^22,23^, and our metaproteome further suggested that these two MAGs also expressed several enzymes involved in fermentation of starch-derived sugars (i.e. glycolysis, **Figure 4**). It should also be noted that a higher number of proteins aligned to those predicted for both UBA1213 and BOa compared to their cultivated *Ruminococcus* relatives (**Figure 1**). Besides *F. succinogenes* and *Butyvibrio fibrisolvens*, several MAGs (i.e. UBA1229, UBA1233, UBA1240 at high levels and ‘APb’, UBA1225 and UBA1258 at lower levels) also displayed significant protein detection levels of GH94, suggesting that cellobiose phosphorylation mediated through the action of GH94 is widespread amongst the rumen microbiome.

**Text S2: Material and Methods**

**Metaproteomics - protein extraction and mass spectrometry**

Proteins were extracted from bulk rumen fluid and different fractions of the solid rumen-incubated biomass. Solid biomass was ground using a Biopulverizer (Biospec, Bartlesville, OK) and liquid nitrogen. SIGMAFAST protease inhibitor was added to prevent protein degradation during sample preparation. Protein concentrations were determined using the bicinchoninic acid (BCA) protein assay (ThermoFisher Pierce, Waltham, MA). Urea and dithiothreitol (DTT) were added to all samples to a final concentration of 8 M and 10 mM, respectively and incubated at 60°C for 30 minutes to denature and reduce proteins. Protein digestion was performed at 37°C (235 rpm) for 3 hours after CaCl_2_ trypsin was added to a 1 mM final concentration and in a 1:50 trypsin:protein (w/w) ratio, respectively. After sample clean-up and concentration, samples were analyzed by reversed phase LC-MS/MS using a Waters nanoACQUITY^TM^ UPLC system (Millford, MA) coupled with an Orbitrap Velos mass spectrometer (Thermo Fisher Scientific, San Jose, CA). The obtained MS/MS scans were subsequently analyzed using MaxQuant^24^ v.1.6.0.13, and proteins quantified using the MaxLFQ^25^ algorithm implemented in MaxQuant. Peptides were identified by searching the MS/MS datasets against the reference databases. To identify common contaminants introduced during sample preparation, this database was complemented with common contaminants, such as human keratin and bovine serum albumin, as well as with reversed sequences in order to estimate the false discovery rate. Tolerance levels for peptide identifications were 6 ppm and 0.5 Da for MS and MS/MS, respectively, and two missed cleavages of trypsin were allowed. Carbamidomethylation of cysteine residues was used as a fixed modification, while oxidation of methionines and protein N-terminal acetylation were used as variable modifications.

**Metatranscriptomics - total RNA extraction and Poly(A) mRNA purification**

For total RNA isolation, frozen rumen-incubated biomass (switchgrass) was manually ground to powder in the presence of liquid nitrogen and immediately added to TRIzol reagent (Invitrogen, Carlsbad, CA). Next, the biomass/TRIzol mixture was transferred into a 2 mL microcentrifuge tube containing Lysing Matrix E (MP Biomedicals Solon, OH), followed by bead beating (3 x 1 min at room temperature, 2 min at 4°C between individual beating steps) using a Mini-Beadbeater-16 (Biospec Products, Bartlesville OK). Homogenized samples were centrifuged (12 000 x *g*, 10 min at 4°C); the supernatant was transferred to new tubes and incubated at room temperature for 5 min. Subsequent TRIzol-based RNA isolation was performed according to manufacturer’s instructions. Poly(A) mRNA, representing transcripts of eukaryotic origin was isolated from total RNA with MicroPoly(A)Purist kit (Invitrogen, Carlsbad, CA) following the manufacturer's instructions. The prepared libraries were quantified using KAPA Biosystem’s next-generation sequencing library qPCR kit and run on a Roche LightCycler 480 real-time PCR instrument. The quantified libraries were then multiplexed, and the library pool was then prepared for sequencing on the Illumina HiSeq platform utilizing a TruSeq paired-end cluster kit, v3, and Illumina’s cBot instrument to generate a clustered flow cell. Sequencing was performed on the Illumina HiSeq2000 using a TruSeq SBS sequencing kit, v3, following a 2x150 indexed run recipe. Adapter sequences and low-quality reads (Q < 10) were trimmed and the reads were further filtered to remove process artifacts using BBDuk included in BBTools^26^ from JGI. After trimming and filtering, human and ribosomal RNA reads were removed by mapping sequences against a modified Silva database^27^ using BBMap^26^. Cleaned reads were combined and the metatransciptome was assembled using MEGAHIT^28^ v.0.2.0.

**Supplementary References**:

1 Hess M, Sczyrba A, Egan R, Kim T-W, Chokhawala H, Schroth G *et al.* Metagenomic Discovery of Biomass-Degrading Genes and Genomes from Cow Rumen. *Science (80- )* 2011; **463**: 463–467.

2 Paez-Espino D, Chen IMA, Palaniappan K, Ratner A, Chu K, Szeto E *et al.* IMG/VR: A database of cultured and uncultured DNA viruses and retroviruses. *Nucleic Acids Res* 2017; **45**: D457–D465.

3 Emerson JB, Roux S, Brum JR, Bolduc B, Woodcroft BJ, Jang H Bin *et al.* Host-linked soil viral ecology along a permafrost thaw gradient. *Nat Microbiol* 2018; **3**: 870–880.

4 Solden LM, Naas AE, Roux S, Daly RA, Collins WB, Nicora CD *et al.* Interspecies cross-feeding orchestrates carbon degradation in the rumen ecosystem. *Nat Microbiol* 2018; **3**. doi:10.1038/s41564-018-0225-4.

5 Anderson CL, Sullivan MB, Fernando SC. Dietary energy drives the dynamic response of bovine rumen viral communities. *Microbiome* 2017; **5**: 155.

6 Gilbert RA, Kelly WJ, Altermann E, Leahy SC, Minchin C, Ouwerkerk D *et al.* Toward Understanding Phage:Host Interactions in the Rumen; Complete Genome Sequences of Lytic Phages Infecting Rumen Bacteria. *Front Microbiol* 2017; **8**: 2340.

7 Markine-Goriaynoff N, Gillet L, Van Etten JL, Korres H, Verma N, Vanderplasschen A. Glycosyltransferases encoded by viruses. *J Gen Virol* 2004; **85**: 2741–2754.

8 Romero P, Bartual SG, Schmelcher M, Glück C, Hermoso JA, Loessner MJ. Structural insights into the binding and catalytic mechanisms of the Listeria monocytogenes bacteriophage glycosyl hydrolase PlyP40. *Mol Microbiol* 2018; **108**: 128–142.

9 Porter CJ, Schuch R, Pelzek AJ, Buckle AM, McGowan S, Wilce MCJ *et al.* The 1.6 Å Crystal Structure of the Catalytic Domain of PlyB, a Bacteriophage Lysin Active Against Bacillus anthracis. *J Mol Biol* 2007; **366**: 540–550.

10 Mizuno CM, Guyomar C, Roux S, Lavigne R, Rodriguez-Valera F, Sullivan MB *et al.* Numerous cultivated and uncultivated viruses encode ribosomal proteins. *Nat Commun* 2019; **10**: 752.

11 Brum JR, Ignacio-Espinoza JC, Kim EH, Trubl G, Jones RM, Roux S *et al.* Illuminating structural proteins in viral ‘dark matter’ with metaproteomics. *Proc Natl Acad Sci U S A* 2016; **113**: 2436–2441.

12 Gilmore SP, Henske JK, O’Malley MA. Driving biomass breakdown through engineered cellulosomes. *Bioengineered* 2015; **6**: 204–208.

13 Artzi L, Bayer EA, Moraïs S. Cellulosomes: bacterial nanomachines for dismantling plant polysaccharides. *Nat Rev Microbiol* 2017; **15**: 83–95.

14 Bayer EA, Kenig R, Lamed R. Adherence of Clostridium thermocellum to cellulose. *J Bacteriol* 1983; **156**: 818–27.

15 Ben David Y, Dassa B, Borovok I, Lamed R, Koropatkin NM, Martens EC *et al.* Ruminococcal cellulosome systems from rumen to human. *Environ Microbiol* 2015; **17**: 3407–3426.

16 Israeli-Ruimy V, Bule P, Jindou S, Dassa B, Moraïs S, Borovok I *et al.* Complexity of the Ruminococcus flavefaciens FD-1 cellulosome reflects an expansion of family-related protein-protein interactions. *Sci Rep* 2017; **7**: 42355.

17 Haitjema CH, Gilmore SP, Henske JK, Solomon K V., de Groot R, Kuo A *et al.* A parts list for fungal cellulosomes revealed by comparative genomics. *Nat Microbiol* 2017; **2**: 1–8.

18 Nagy T, Tunnicliffe RB, Higgins LD, Walters C, Gilbert HJ, Williamson MP. Characterization of a Double Dockerin from the Cellulosome of the Anaerobic Fungus Piromyces equi. *J Mol Biol* 2007; **373**: 612–622.

19 Shoham Y, Lamed R, Bayer EA. The cellulosome concept as an efficient microbial strategy for the degradation of insoluble polysaccharides. *Trends Microbiol* 1999; **7**: 275–281.

20 Youssef NH, Couger MB, Struchtemeyer CG, Liggenstoffer AS, Prade RA, Najar FZ *et al.* The genome of the anaerobic fungus orpinomyces sp. strain c1a reveals the unique evolutionary history of a remarkable plant biomass degrader. *Appl Environ Microbiol* 2013; **79**: 4620–4634.

21 Dai X, Tian Y, Li J, Luo Y, Liu D, Zheng H *et al.* Metatranscriptomic analyses of plant cell wall polysaccharide degradation by microorganisms in the cow rumen. *Appl Environ Microbiol* 2015; **81**: 1375–86.

22 Kuchtová A, Janeček Š. Domain evolution in enzymes of the neopullulanase subfamily. *Microbiology* 2016; **162**: 2099–2115.

23 Rumbak E, Rawlings DE, Lindsey GG, Woods DR. Characterization of the Butyrivibrio fibrisolvens glgB gene, which encodes a glycogen-branching enzyme with starch-clearing activity. *J Bacteriol* 1991; **173**: 6732–6741.

24 Cox J, Mann M. MaxQuant enables high peptide identification rates, individualized p.p.b.-range mass accuracies and proteome-wide protein quantification. *Nat Biotechnol* 2008; **26**: 1367–72.

25 Cox J, Hein MY, Luber CA, Paron I, Nagaraj N, Mann M. Accurate Proteome-wide Label-free Quantification by Delayed Normalization and Maximal Peptide Ratio Extraction, Termed MaxLFQ. *Mol Cell Proteomics* 2014; **13**: 2513–2526.

26 BBMap – Bushnell B. – sourceforge.net/projects/bbmap/. .

27 Pruesse E, Quast C, Knittel K, Fuchs BM, Ludwig W, Peplies J *et al.* SILVA: a comprehensive online resource for quality checked and aligned ribosomal RNA sequence data compatible with ARB. *Nucleic Acids Res* 2007; **35**: 7188–7196.

28 Li D, Liu C-M, Luo R, Sadakane K, Lam T-W. MEGAHIT: an ultra-fast single-node solution for large and complex metagenomics assembly via succinct de Bruijn graph. *Bioinformatics* 2015; **31**: 1674–1676.

**Text S3: FASTA sequences**

**(A)** FASTA sequences of the viral proteins detected in the metaproteome, and annotated as putative auxiliary proteins (three bacterial extracellular solute-binding proteins and one oxidoreductase), and a viral protein sequence detected at the upper range of the protein detection level:

Bacterial extracellular solute-binding proteins (3):

>Vir_gene_id_63857

GDQGTRAIINNLYGGTFTNPEHTAYTADSPENIKALELLQSLEGVYFNAAENGGEEITAFRQGLLKMAFCWNIAQQLPGETTGDRTYDDEEIVFMSFPAEDGVAALCGGIWGFGIFDNGDAAKIEAAKEFIKYFCDGAGTEAAVKTAKYFAVRDVADGKDISGIWADDDTMNAYKVLMPYLGDYYQVTSGWATARTEWWNMLQRIGDGGDVATEVAVFCANANAAAAA

>Vir_gene_id_63859

MLTLSExxxxLWTYPIGQWGNADAVGALMADFEAATGIKVTVEYLDYTNGDDQVNMAIEGKNAPDLVMEGPERLVANWGAKGYMVDLADLWDEEDLAQVNPSCVSACFTADGACYEYPV

>Vir_gene_id_63912

MKKVLALVLAMVMVLSLVSFASADDKVISIFLGGGTPLSMDPALNSASAGSNIIRSAFAGLTGFQYNEAGEPEMAPEIAESYEVSEDGLTYSFVLRENLKWSDGTDCTASQIKASWERAASAELGADYGFLYDVISRAEDGSLAIDVDDAARTFVVHLPQPCAYFLDLCAFVPFYPVRVDLADNEGIWATNPETYVGLGAFKMTKYAVDDVISFEKNPYYWNADAVKLSGLNFYLSEDNTAILTAYENGTVQYIQSISSSEFDRLNATYPGELAFWPTQGTYYILFNVHKDLSPASKQLTVQEQSKARFALGELINRYEVVTYVTKGGEVAATGFFPAGLADGLNSDVRAAEGYGVWYTGTNEFSDVNPDYTVDQVEALQTLVDLGYPYTGSIEGGDIVFTDFPSIEFAFNNSGNNALIIQYVQETWNQFGITGVVNQEAWATLQSKLKAGDAEAARMGWIADFNDVVNFLEIFISASGNNYPRLGREIGDYTRNSEVTADAGKGAYWGPNGDQTWAEAYDALVDAVKAATDPVERASLAAEAEKVLMATGGVNPLYYYTTAQMLKPNVTDVIRLATGDVIWTYADIN

Oxidoreductase (1):

>Vir_gene_id_71822

IYALEHGIPVLLEKPFSVTLEEAEAVCRAEKASGKFVSVGFQPRFDANMQMIKKIVDSGVLGKIYYIQTGGGRRRGIPGSTFIEKSTGGIGALGDIGCYSLDMVLNAIGYPKPLTVSGYISDFFGKNPKYNGKDAERFSVDDFAAAFIRLEGDIILDFRIAWAMHVNTPGDTIIFGTEGALRIPSTDCxxxxRSRGSPTG

Putative porter protein (high protein detection level; log_2_(LFQ) score = 31.5) (1):

>Vir_gene_id_42007

MWSAIVQKIKEILQKMIGKNTLEQTLHVSPVISSEMENAIQLWGDMYKGNPPWVHEPTWEDPSRVVSLGIPALVASEKARTALLEFSSEVTTPIEEVEVQNPNYQPPMQDEFGVIKPEIGTPTVKESKPVGNTERADFLNENYKWLKKRLRTQIEYGIAKGGLVIKPYLIQRKGEWAFEYDYIQADAFYPLSFDNNGDITEAAFLQTHKDKEFVYTRVEYHKWVDNVVTVINRAFKTTANVHDTNGIDLGMEVPLTEVNEWKDLEPTTTITDVDRPLFAYFKMPEANTIDTTSSLGVSGYSRAVQLIRDADEQYSRLLWEYEGGEMAIDVDRDALKFIEGSDGTVHTVLPKLQQRLFRKIDLGNDDVYNPFAPTLRDAQYIQGLNAILMRIEDVTGLSRGTLSDVTIEAKTATELKMLKQRSYQTNEHIQEAIQTTLEDVVYIMNVYCDLYDVTPDGEYEISFEWDDSLITDKDDELGKRITLQQNGLASRVENRMWYFGETERQAMEALAKIDEENAKQAMANMELEQSSQALHENNNGNTTGMNNETPNKNQPNKIKGVDKNNAQ

**(B)** FASTA sequences of detected proteins annotated as GH48 in MT-eukDB.

>contig_51_8776357_length_1487_multi_6_in_0_out_0.p1

GQNWCWQIGCSHAHEFYQNPLAAYALITELKSGMKAEGAAKDYETSLTRQMEFYQWLQSANGPIAGGATNSYKGRYEAYPAGSSTFYGMVYTPHPVYADPGSNHWIGNQVWAVQRLAELFYWERKDNNQTIVSPGGVSLDKALEDILDKWVAWFVNNSVLTKDGECYMPSNLDWSGQPDKWNNAAPTNSGLTCEITGYGNGDTGCLSSLSNTLIYYAKAKGVKVADIAKYDETAVGGEYKSGIDDVTPLAGAKSYKTGDAEVPAVGLYLAQQIMDRTWNNARDDIGLTRVDHNGSLARMFSQPVHVPKAYNGTMPSGDTIANGATFLSLRSMYTDSSKCKGVKTSDEALKLVQTLKDAYDKDEAAGAKWSGGYSASSAEGKAELEKFTNVAAVELKYHRFWHMGDAMMALGTLAELYPDLEPTKGNSGTDPTDPKPGSVTLWGDADESGTVDILDVIQVNKFLLGVAKLSAQGALNADLDQSGDPDSTDSLNILK

>contig_81_3781524_length_1924_multi_3_in_0_out_0.p1

SEGREYLLHWLADVDDWYGFSGSARGEKGEFTFINTFQRGDQESCFETIPHPSIETLKYGNSNQGMKFAFQKSTAKSWSYTNAPDAEDRAIQAAYAANRWGVSNSVSSKAGMMGDFCRNDMYDKYYKAIGCQSMQTDASGGSGDKGKHYLMSWYTAWGGAEDGTWAWQIGCSHAHQFYQNPLAAYGLLYGDLPMEADGAKKDYETSLERQMEMYLWLSSIEGPFAGGCTNSWMGAYEKYPSGVPTFHDMAYIEQPVYADPGSNGWTGNQYWATQRLAELYYVVKTDSDKSSVKPGGLSLEKALETVLDKWVDFFVTNSKLTDDGDYEVPSGLTWEGAPDDWTGSYKENSNLHAKLNGMQNTDLGCVCSLANTLIYYAAANGVEASAASKEGTALPEKALYLANQLIDRVWQKGRDDIGCSRTEHNGSLARFWAQEVYAGGGSGEYPYGYTVKDGSTFVDIRPMYADEGQAYSELYAELKAAYDKDVANGAKIKEVDTTSNYKYGSDCRTTNEDFKNVEAVDLNYHRFWHAGDVMMANGAMALLYPDVTPGTTPAPETTPAPTTTTTAAPVTTPAPETTPAPSTAEIKAKLYGDTNCDDKVDVSDAVLLAKYLTADASGSVSEQGRANANVISGTLDGEDLS

>contig_121_898400_length_3120_multi_7_in_0_out_0.p1

MLPKKTKAVVASILTAAMTTNVIAATVVPASAARTKANKYGDSTYAQRFMSMYDDVITNGQTNGYLSKNDGGSGSFGVPYHAREELIVEAPDYGHETTSEAMSYLVWVAAMHDNIVKNSGEKFSGASTNDLAKAWKTMEVMIPDVQDNFWQASSVSSQYCGEYDTPDQCPNAWAGESSKTAENPIFNKFTSVYQGKNGNGGLYLMHWLADVDNWYGFGSGTEFTFINTFQRGEQESCWETVPFPCVEEKKYGNSQQGLKGIFNRDSNVTAQWAYTNAPDAEDRAIQGVYDAIQWKVADSSVTAKASEMGDELRNNMYDKYYQEISTNTSWSNGNAGDKSKHYLMNWYTSWGGALKSTGQNWCWQIGCSHAHEFYQNPLAAYGLLTSMNMKADGAKQDYTKSLERQLEFYLWLQSSNGPIAGGATNSYKGRYLSYPSGVPTFYGMMYVEHPVYADPGSNHWTGNQVWAVQRLAELYYWVKKNGDNTGVRPGGMSMEAALEQILDKWCAWFVNNTILTSDGDFYMPSNLDWSGAPDSWNGSATSNSGLTCKITGYGNTDLGCISSLANTLTYYAKAKGVKASDISGLTESSVGGSFNFNIEGASNAKRGSKTYSANDSALPKASLFLAKSLIDRAWNKGRDNLGMSRTEHNGSMARFFSQEVYIPESYNGTMPNGDTLKNGATFESIRTMYNGNCAGAETSSECVKLVEEMRAAYKKDVANGAKWSNKYSASDSEGQAELAKFKNVANVDLNYHRFWHAGDDMMAMGVMATLYPDMKPGIIDDDHHDDDTTKTDYPKNVKANYNTQYHQIQFVWDKVSGADRYGIAVYLAGKWRVQTSNITTNSYVTPKNLTPGMSYKVAVAARVNGKWNTTDPIK

>contig_91_6181101_length_2510_multi_9_in_0_out_0.p1

MISKKNKALAAGILAAAMSASAVIPSMASAAEKSSYAKETTYADMFASLYDDVVTNGQTNGYLSKQTNGSGFGIPYHSVETFIVEAPDYGHETTSEAMSYIVWMAAMHDVLAANNVISGSKGDLAKAWNTMEAMIPGWSKASGRDTEIKYSSIWDQKDGLKADTSEECDSPEDYPAKQPGVKAENPIYDTFKSAYSSDNGYYLMNWLADVDDWYGFSKETKGEGKFTFINTFQRGEQESCFETVPAPCLEELKWGMKSTDKDNGNGIKAIFNGKDKVPSQYSFTNAPDAEDRCIQAVHFANMYNAGDSKVSGLAGKMGDQCRNDMFDKYYKKIAVDTKISDSSAGMDSKHYLMSWYTAWGGALTASYGDYGWAWQIGCSHSHQFYQNPLAAYALLYDSDITAGMKAKDAESDYKESLKRQIEMYQWLQSVDGPFAGGCTNSWRGRYEEYPSGHATFYDMAYVPHPVYADPGSNHWIGNQVWSTQRLAELYYYVVTNGDKSGQKYGGLDLETALDKLLERWIAWFEKNTQFNYEDEDGNTHTYAIPSTLDWGSSDTDYTCTPDTWTGTYDENGNQKLTCKIGGYGQGDVGCVSSLCNTLIYYAKAKNVDSKFAKQEGTSVAEKALYLANRLLTAQYEEGRDEIGITFTDCNPSLKRVFEEKVYIPDFYSGEMPDGSKLEPGATFSSIRKSYEQDSMWQEAQKYWKGEGTDNNKDGTVDIKDFQFQYHRFWHAGDAVVAYGTMALLFPEVTPGIDGETTTQPATQPATQPATQPATQPATQPATQPATQPATQPATSGKVTKWGDANCDGDVDMADAVLIMQSLA
